# Comparative efficacy and safety of alpha-blockers as monotherapy for benign prostatic hyperplasia: a systematic review and network meta-analysis

**DOI:** 10.1038/s41598-024-61977-5

**Published:** 2024-05-15

**Authors:** Beema T Yoosuf, Abhilash Kumar Panda, Muhammed Favas KT, Saroj Kundan Bharti, Sudheer Kumar Devana, Dipika Bansal

**Affiliations:** 1 Department of Pharmacy Practice, National Institute of Pharmaceutical Education and Research (NIPER), S.A.S. Nagar, Mohali, Punjab India; 2 Department of Urology, Postgraduate Institute of Medical Education and Research, Chandigarh, India

**Keywords:** Benign prostatic hyperplasia, International prostate symptom score, Network meta-analysis, Quality of life, Health care, Medical research, Urology

## Abstract

Despite the availability of various drugs for benign prostatic hyperplasia (BPH), alpha(α)-blockers are the preferred first-line treatment. However, there remains a scarcity of direct comparisons among various α-blockers. Therefore, this network meta-analysis (NMA) of randomized controlled trials (RCTs) aimed to evaluate the efficacy and safety of α-blockers in the management of BPH. A comprehensive electronic search covered PubMed, Embase, Ovid MEDLINE, and Cochrane Library until August 2023. The primary endpoints comprised international prostate symptom score (IPSS), maximum flow rate (Qmax), quality of life (QoL), and post-void residual volume (PVR), while treatment-emergent adverse events (TEAEs) were considered as secondary endpoints. This NMA synthesized evidence from 22 studies covering 3371 patients with six kinds of α-blockers with 12 dose categories. IPSS has been considerably improved by tamsulosin 0.4 mg, naftopidil 50 mg and silodosin 8 mg as compared to the placebo. Based on the p-score, tamsulosin 0.4 mg had the highest probability of ranking for IPSS, PVR, and Qmax, whereas doxazosin 8 mg had the highest probability of improving QoL. A total of 297 adverse events were reported among all the α-blockers, silodosin has reported a notable number of TEAEs. Current evidence supports α-blockers are effective in IPSS reduction and are considered safer. Larger sample size with long-term studies are needed to refine estimates of IPSS, QoL, PVR, and Qmax outcomes in α-blocker users.

## Introduction

Benign prostatic hyperplasia (BPH) is a ubiquitous urological disease that inevitably affects older men, occurring in up to 50% of men over 50 to 60 years, rising to 90% by age 80, and its predominance increases further with age^1–3^. BPH results from the noncancerous prostate gland enlargement induced by cellular hyperplasia of both glandular and stromal components^4^. Numerous sources of evidence reveal that in addition to ageing and family history, modifiable risk factors such as enlarged prostate, dyslipidemia, hypertension, hormonal imbalance, obesity, metabolic syndrome, diet, alcohol use, and smoking can collectively contribute to BPH^5,6^. Many individuals with BPH experience lower urinary tract symptoms (LUTS) in the form of irritative (frequency, nocturia and urgency) and obstructive urinary symptoms (hesitancy, intermittency, weak stream, incomplete bladder emptying and acute urinary retention (AUR))^1^. LUTS correlated with BPH drastically compromises the quality of life (QoL), primarily disrupting sleep and daily activities^7^. Ipso facto, the intent of BPH treatment is to alleviate these troublesome and irritating symptoms^1^.

Pharmacological management of LUTS correlated with BPH has emerged over the last 25 years^6^. Existing medical therapy for BPH includes alpha-adrenergic receptor antagonists (α-blockers), anticholinergics, 5-alpha reductase inhibitors (5-ARIs) and phosphodiesterase inhibitors (PDE5-Is). Medical therapy is generally considered the initial treatment option for patients with moderate to severe LUTS while surgical approaches like transurethral resection of the prostate (TURP) are recommended for patients who had poor response to medical therapy or those with specific indications like refractory urinary retention, recurrent hematuria and those with severe bladder outlet obstruction leading to hydroureteronephrosis^4^. α-blockers are considered as the first-line drugs for treating BPH. Long-acting α-blockers, such as doxazosin, terazosin, tamsulosin, alfuzosin and silodosin, have been approved by the Food and Drug Administration (FDA) for the treatment of BPH^8^. They can mitigate symptoms by blocking endogenously secreted noradrenaline on smooth muscle cells in the prostate gland, thus reducing prostate tone and bladder outlet obstruction^9^.

Despite the fact that a large number of drugs are now available to treat BPH, α-blockers have a significant impact on improvement in International Prostate Symptom Score (IPSS), maximum flow rate (Qmax), post-void residual (PVR) and QoL^8,10,11^. Even though several clinical trials have been performed to explore the effectiveness of α-blockers for BPH, direct comparisons among these drugs are still lacking and there is conflicting information coming forward from meta-analysis^12–14^. For instance, a network meta-analysis (NMA) conducted on drug therapies for BPH assessed the effectiveness of multiple drug classes, instead of individual agents^15^. Furthermore, the most recently published NMA demonstrates merely IPSS, peak urine flow rate (PUF), and adverse events (AEs) among mono-drug therapies for LUTs related to BPH^16^. At present, none of the NMA have extensively evaluated the efficacy of these agents within the class in terms of the majority of outcomes as well as treatment-emergent adverse events (TEAEs). Therefore, the aim of the present study is to address the knowledge gap surrounding the comparative effectiveness of α-blockers for BPH based on available randomised controlled trials (RCTs) and rank these agents for clinical consideration.

## Methods

This network meta-analysis (NMA) was executed following the Preferred Reporting Items for Systematic Reviews and Meta-Analyses (PRISMA) extension statement for NMA. We have applied frequentist network meta-analysis for its simplicity associated with the model formulation^17^. The protocol was registered in the Prospective Register of Systematic Reviews (CRD42022365398).

### Literature searches

A comprehensive electronic search of PubMed, Ovid MEDLINE, EMBASE and the Cochrane library, was carried out to identify the eligible studies. Additionally, a manual search in Google Scholar was performed. The initial search strategy was developed in the PubMed database, and the search strings used for electronic searches consist of combinations of keywords and medical subject headings (MeSH) terms like “alpha-blockers”, “Alfuzosin”, “Tamsulosin”, “Doxazosin”, “Terazosin”, “Silodosin”, “Naftopidil”, “Benign prostatic hyperplasia” and “Randomised controlled trial”. A methodological search filter was adopted to identify RCTs, and the search was limited to English-language publications. This search strategy serves as a template for alternative search algorithms customized to different databases, such as EMBASE, Ovid MEDLINE, and the Cochrane Library. In addition, the reference lists of the selected studies and review articles were hand-searched for additional potentially pertinent studies.

### Study eligibility

This systematic review and NMA sought studies that met the PICO (P—population, I—intervention, C—comparator, O—outcome) framework. RCTs that investigated the efficacy and safety of α-blocker in men aged 45 and above with LUTS related to BPH were included. However, monotherapy with α-blockers were eligible, including selective (i.e., terazosin and doxazosin) and uroselective (tamsulosin, silodosin, alfuzosin and naftopidil), with no restrictions on α-blocker dosage^18^. As the research question also explored placebo-controlled trials, therefore the placebo serves to be the comparator. The key outcomes of interest were IPSS, QoL, PVR and Q max. TEAEs are also evaluated in order to provide a comprehensive overview of these drugs. Reviews, editorials, case reports, conference abstracts, studies that deviated from the aimed outcomes or with incomplete results and articles published in non-English were excluded.

### Study screening

Two reviewers (BY and AP) worked independently to screen citations and evaluate full-text records for eligibility. Initially, only the title and abstract were screened, and the full texts of presumably pertinent articles were subsequently assessed for ultimate inclusion. A cross-check has been performed at both stages to ensure full compliance with eligibility requirements. Disputes regarding the full-text articles were rectified through discussion with a third reviewer (DB).

### Data extraction

Two reviewers (BY and AP) individually extracted the following information into a spreadsheet: study characteristics (Title, first author, publication year, country, duration of treatment), population (study setting, sample size, baseline demographics), characterization of interventions (drug name and dose), and outcomes (reduction in IPSS and PVR, improvement in QOL, Qmax). Disagreements among reviewers were resolved by discussion or, if necessary, communicating with a third reviewer (DB). If any imperative information about study outcomes was missing or unclear in the published studies, the authors were contacted to seek clarification or additional data.

### Risk of bias

The methodological quality of each included RCT was critically appraised employing the revised Cochrane Risk of Bias Tool (ROB 2.0)^19^. This tool captures six main sources of bias, comprising random sequence generation, allocation concealment, missing outcome data, blinding, selective reporting, and other sources of bias. Each domain has been assigned a score of low, moderate to high.

### Statistical analysis

To account for certain methodological and clinical heterogeneity across studies, and to acquire the optimal generalizability in the meta-analytical treatment effects, we adopted a random-effects model^20^. As all the efficacy outcomes are continuous data, the effect size was computed as standardised mean difference (SMD) along with 95% confidence intervals (CI), and the outcome data was compiled using direct and indirect evidence employing a frequentist approach.

Statistical analysis was carried out using the “netmeta” package of R Studio and data were analysed following the intention-to-treat approach. A network plot of interventions was used to visualise the evidence gathered and offered a succinct overview of its characteristics. Direct evidence has gathered by pair-wise meta-analysis, while indirect evidence was obtained through indirect comparisons. The treatments were ranked using p-scores derived from the surface under the cumulative ranking curve (SUCRA). Higher p- scores tend to indicate a higher probability of being the most effective treatment^21^. In order to evaluate inconsistency, both global and local approaches were utilized. Under the presumption of a full design-by-treatment interaction random effects model^22^, the Q test and the I^2^ statistic are adopted to evaluate consistency^23^. The local approach distinguishes indirect from direct evidence (SIDE) using the back-calculation method. The comparison-adjusted funnel plot was utilized to evaluate small-study effects for each outcome with ≥ 10 studies, where the overall treatment effect for every comparison was estimated employing random-effect meta-analysis model^24^. All eligible drugs have been ordered from oldest to newest according to their international market authorisation dates. Furthermore, the Grading of Recommendations Assessment, Development, and Evaluation (GRADE) ratings were deployed to assess the certainty of evidence in networks employing the Confidence In Network Meta‐Analysis (CINeMA) framework^25^.

## Results

### Study selection

**Figure 1:**
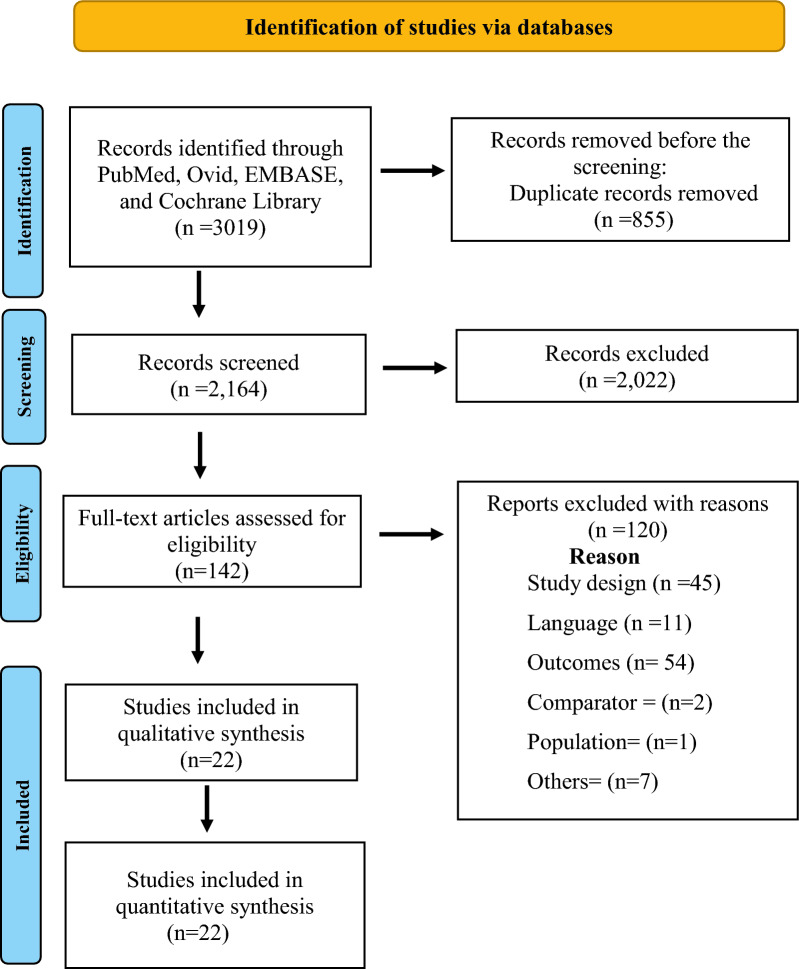
PRISMA flow chart of literature searches and results. (*PRISMA* Preferred Reporting Items for Systematic Reviews and Meta-Analyses).

The literature search of across multiple databases yielded a total of 3019 potentially relevant citations (Table S9). After duplication screening, 2164 articles were found. Of these 2022 articles were removed after the initial title and abstract screening and retrieved 142 articles for full-text review. Finally, 22 RCTs (3271 participants) published from 2000 to 2023 were included (Fig. 1)^12–14,26–44^.

### Study characteristics

The included RCTs comprised the currently used six kinds of α-blockers with different dose categories including Naftopidil 25 mg, 50 mg and 75 mg, Silodosin 8 mg, Tamsulosin 0.2 mg and 0.4 mg, Alfuzosin 2.5 mg and 10 mg, Doxazosin 2 mg, 4 mg and 8 mg, Terazosin 0.5 mg with a total 3,371 participants. Among the 22 included studies, 10 were multi-centric^26,29,31,32,34,36,39–42^, while 12 were single-centric^12–14,27,28,30,33,35,37,38,43,44^. Nine trials were conducted in Japan^26,30–33,36,39,40,43^, five in India^12–14,37,44^, two in Korea^41,42^ and one each in China^34^, Indonesia^29^, Europe^27^, Philippines^28^, Egypt^35^ and Turkey^38^. Most studies (91%) were published after 2005 and, over half of the studies (50%) involved more than 100 patients. A majority of trials (72.73%) had treatment durations of more than 4 weeks. The mean (SD) age of the patients was 65.3 (6.7) years (Table 1). According to IPSS, the symptoms of patients in the included trials varied from moderate to severe, with a baseline mean (SD) of 18.1 (4.6). The baseline mean (SD) value of QOL was 4.2 (0.8), Q max (ml/s) 10.2 (3.4), and PVR (ml) 49.0 (34.2).

**Table 1:** Study characteristics in brief.

Study characteristic	Studies (n = 22)	Studies (%)
**Year of publication**		
2000–2004	2	9.09
2005–2009	5	22.73
2010–2014	7	31.82
2015–2020	8	36.36
**Country**		
Japan	9	40.91
India	5	22.73
Korea	2	9.09
China	1	4.54
Indonesia	1	4.54
Turkey	1	4.54
Philippines	1	4.54
Egypt	1	4.54
Europe	1	4.54
**Location**		
Single-centric	12	54.54
Multi-centric	10	45.45
**Study design**		
Randomized clinical trial	22	100.00
**Sample size**		
0–99	11	50.00
100–199	5	22.73
200–299	3	13.64
299–399	1	4.54
Above 400	2	9.09
**Study duration**		
≤ 4 weeks	6	27.27
5–8 weeks	5	22.73
9–12 weeks	10	45.45
≥ 12 weeks	1	4.54
**Number of interventions**		
2	18	81.81
3	4	18.18
**Outcomes**^a^		
IPSS	22	100.00
QoL	14	63.63
PVR	18	81.81
Qmax	16	72.72
**Risk of bias**		
Low risk	15	68.18
Some concerns	3	13.64
High risk	4	18.18

^a^As some studies report more than one outcome, the values in this category do not match the totals.

IPSS, International prostate symptom score; Q max, maximum flow rate; QoL, quality of life; PVR, post-void residual.

In terms of study quality, 15 trials (68.18%) exhibited a low risk of bias, three trials (13.64%) had a moderate risk of bias, and four trials (18.18%) had a high risk of bias (Table 2).

**Table 2:** Study characteristics in detail.

Study	Country	Study design	Location	Study duration (weeks)	Age; mean (SD)	Total participants (N)	Intervention arms: number of patients	IPSS mean (SD)	QoL mean (SD)	Qmax mean (SD)	PVR mean (SD)	Risk of bias
Okada et al.^25^	Japan	SB, RCT	MC	4	65.7 (8.2)	61	Terazosin 1 mg: 31 patients	18.8 (6.0)	4.4 (1.1)	8.5 (2.0)	NR	SC
							Tamsulosin 0.2 mg: 30 patients	20.6 (7.0)	4.7 (1.0)	8.1 (2.5)	NR	
Kerrebroeck et al.^26^	Europe	DB, RCT	SC	12	64.6 (7.6)	447	Alfuzosin 2.5 mg: 150 patients	16.8 (3.7)	3.3 (1.0)	8.8 (1.9)	NR	LR
							Alfuzosin 10 mg: 143 patients	17.2 (3.5)	3.3 (0.9)	9.3 (1.9)	NR	
							Placebo: 154 patients	17.8 (4.3)	3.3 (1.0)	9.1 (2.0)	NR	
Lapitan et al.^27^	Philippine	DB, RCT	SC	8	62.8 (9.1)	76	Tamsulosin 0.2 mg: 40 patients	21.6 (5.3)	NR	9.1 (2.4)	27.8 (64.6)	LR
							Alfuzosin 10 mg: 36 patients	22.6 (5.5)	NR	9.4 (2.7)	14.9 (21.2)	
Rahardjo et al.^28^	Indonesia	OL, RCT	MC	6	64.7 (6.3)	101	Tamsulosin 0.2 mg: 50 patients	18.6 (4.2)	NR	9.9 (3.3)	43.9 (24.4)	HR
							Doxazosin 2 mg: 51 patients	18.8 (4.2)	NR	10.6 (3.1)	46.3 (32.3)	
Yokoyama et al.^29^	Japan	RCT	SC	4	69.9 (6.1)	139	Naftopidil 25 mg: 72 patients	18.8 (6.5)	4.7 (1.0)	8.8 (2.7)	26.4 (33.8)	LR
							Naftopidil 75 mg: 67 patients	19.3 (5.5)	4.7 (0.8)	8.6 (3.1)	28.1 (38.7)	
Ukimura et al.^30^	Japan	RCT	MC	8	69.2 (7.5)	59	Naftopidil 50 mg: 31 patients	17.2 (6.4)	4.7 (1.0)	9.9 (5.5)	19.3 (26.1)	HR
							Tamsulosin 0.2 mg: 28 patients	18.9 (6.6)	4.8 (1.2)	9.6 (4.8)	19.6 (20.2)	
Masumori et al.^31^	Japan	RCT	MC	12	64.9 (7.6)	95	Naftopidil 50 mg: 48 patients	15.0 (5.9)	4.2 (1.0)	10.5 (5.4)	57.7 (54.9)	LR
							Tamsulosin 0.2 mg: 47 patients	17.8 (5.7)	4.7 (0.7)	11.0 (4.2)	58.8 (66.5)	
Yokoyama et al.^32^	Japan	RCT	SC	12	70.3 (1.1)	136	Silodosin 4 mg: 45 patients	18.7 (0.7)	4.5 (0.1)	9.0 (0.6)	57.6 (6.9)	SC
							Tamsulosin 0.2 mg: 45 patients	18.0 (1.1)	4.5 (0.1)	8.5 (3.4)	29.7 (5.5)	
							Naftopidil 50 mg: 46 patients	17.4 (0.8)	4.5 (0.1)	8.6 (0.6)	39.1 (7.7)	
Zhang et al.^33^	China	OL, RCT	MC	8	68.6 (8.3)	200	Doxazosin 4 mg: 100 patients	19.5 (5.7)	4.2 (0.9)	9.1 (2.4)	29.2 (27.8)	LR
							Tamsulosin 0.2 mg: 100 patients	18.4 (4.5)	3.9 (0.8)	9.6 (2.7)	25.9 (24.9)	
Shelbaia et al.^34^	Egypt	SB, RCT	SC	52	53.9 (6.1)	60	Tamsulosin 0.4 mg: 30 patients	13.3 (0.6)	NR	NR	NR	LR
							Placebo: 30 patients	15.0 (0.8)	NR	NR	NR	
Yamaguchi et al.^35^	Japan	RCT	MC	12	69.7 (7.4)	97	Silodosin 8 mg: 53 patients	16.9 (5.5)	4.6 (0.9)	10.4 (5.0)	42.9 (52.5)	LR
							Naftopidil 75 mg: 44 patients	18.9 (7.0)	4.8 (0.9)	9.9 (5.3)	59.9 (62.0)	
Kumar et al.^36^	India	RCT, PC	SC	2	65.2 (8.7)	34	Silodosin 8 mg: 23 patients	25.7 (2.5)	NR	12.4 (5.6)	80.0 (36.0)	LR
							Placebo: 11 patients	24.9 (1.8)	NR	8.6 (5.8)	110.0 (25.0)	
Pande et al.^37^	India	SB, RCT	SC	12	62 (7.7)	61	Silodosin 8 mg: 32 patients	18.4 (3.3)	NR	NR	49.2 (50.3)	LR
							Tamsulosin 0.4 mg: 29 patients	18.4 (3.9)	NR	NR	58.9 (65.6)	
Perumal et al.^14^	India	RCT	SC	4	60 (5.3)	60	Naftopidil 50 mg: 30 patients	20.0 (2.5)	NR	NR	99.2 (12.3)	SC
							Tamsulosin 0.4 mg: 30 patients	21.3 (2.8)	NR	NR	102.9 (10.8)	
Keten et al.^38^	Turkey	RCT	SC	12	59.8 (7.6)	162	Doxazosin 4 mg: 63 patients	19.1 (5.4)	4.6 (0.9)	11.9 (2.5)	40.2 (22.4)	LR
							Doxazosin 8 mg: 44 patients	20.4 (5.2)	4.3 (1.1)	11.6 (2.3)	45.5 (27.4)	
Seki et al.^39^	Japan	OL, RCT	MC	12	71.4 (8.5)	268	Silodosin 4 mg: 133 patients	20.5 (6.1)	4.9 (0.9)	9.1 (3.8)	54.8 (60.9)	HR
							Silodosin 8 mg: 135 patients	19.4 (6.0)	4.8 (0.9)	10.7 (4.4)	46.0 (48.9)	
Manohar et al.^12^	India	DB, RCT	SC	12	58.2 (8.4)	269	Tamsulosin 0.4 mg: 89 patients	16.3 (6.1)	2.4 (0.9)	11.6 (2.6)	44.8 (27.1)	LR
							Alfuzosin 10 mg: 87 patients	16.2 (6.5)	2.4 (0.9)	12.7 (2.6)	41.9 (19.3)	
							Silodosin 8 mg: 93 patients	14.3 (5.3)	2.3 (0.8)	12.1 (2.6)	53.6 (29.0)	
Patil et al.^13^	India	PG, RCT	SC	4	65.2 (8.7)	102	Tamsulosin 0.4 mg: 54 patients	14.4 (2.8)	NR	11.3 (1.9)	92.6 (16.3)	LR
							Silodosin 8 mg: 48 patients	14.1 (2.1)	NR	10.8 (1.7)	91.1 (17.2)	
Matsukawa et al.^40^	Japan	OL, RCT	MC	12	70.5 (7.8)	314	Silodosin 8 mg: 157 patients	18.8 (6.2)	4.8 (0.9)	8.2 (3.6)	41.0 (34.0)	HR
							Naftopidil 75 mg: 157 patients	18.9 (6.1)	4.9 (0.9)	8.4 (3.0)	44.0 (37.0)	
Chung et al.^41^	Korea	DB, RCT	MC	12	63.7 (8.7)	494	Tamsulosin 0.4 mg: 162 patients	19.9 (5.0)	4.1 (0.8)	10.4 (2.4)	38.9 (52.2)	LR
							Tamsulosin 0.2 mg: 165 patients	19.9 (4.8)	4.1 (0.8)	10.4 (2.7)	39.1 (44.1)	
							Placebo: 167 patients	19.9 (4.8)	4.1 (0.7)	10.5 (2.9)	37.8 (46.0)	
Kwon et al.^42^	Korea	RCT	MC	8	65.4 (7)	94	Tamsulosin 0.2 mg: 45 patients	19.1 (7.2)	NR	15.5 (8.4)	37.0 (47.6)	LR
							Naftopidil 75 mg: 49 patients	16.9 (6.2)	NR	16.4 (8.3)	34.7 (34.4)	
Matsumoto et al.^43^	Japan	NB, OL, RCT	SC	4	71.6 (6.1)	42	Tamsulosin 0.2 mg: 22 patients	14.9 (7.3)	4.1 (0.9)	NR	NR	LR
							Silodosin 4 mg: 20 patients	15.2 (5.9)	4.5 (1.2)	NR	NR	

RCT: randomised controlled trial; OL-RCT: open label RCT; DB-RCT: double-blind RCT; SB-RCT: single-blind RCT; MC: multi-center; SC: single-center; LR: low risk; HR: high risk; SC: some concerns; NR: not reported.

### Efficacy outcome

#### International prostate symptom score (IPSS)

**Figure 2:**
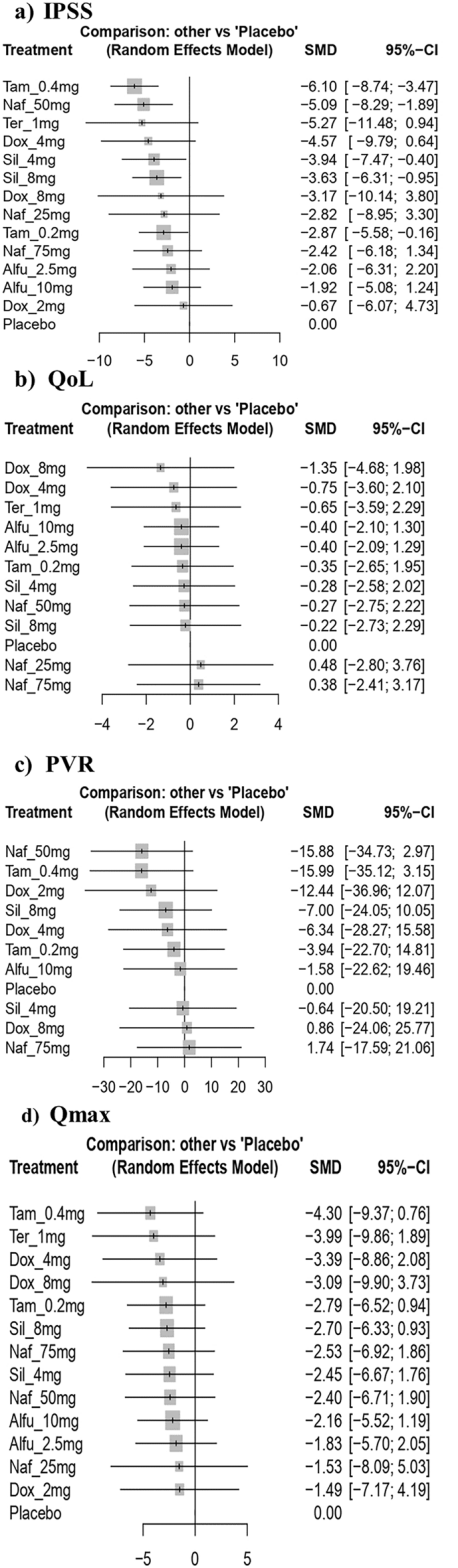
Forest plot of interventions as measured by the international prostate symptom score (IPSS), quality of life (QoL), post-void residual volume (PVR) and maximum flow rate (Q max). Tam = tamsulosin, Alfu = alfuzosin, Naf = naftopidil, Tera = terazosin, Dox = doxazosin, Sil = silodosin.

**Figure 3:**
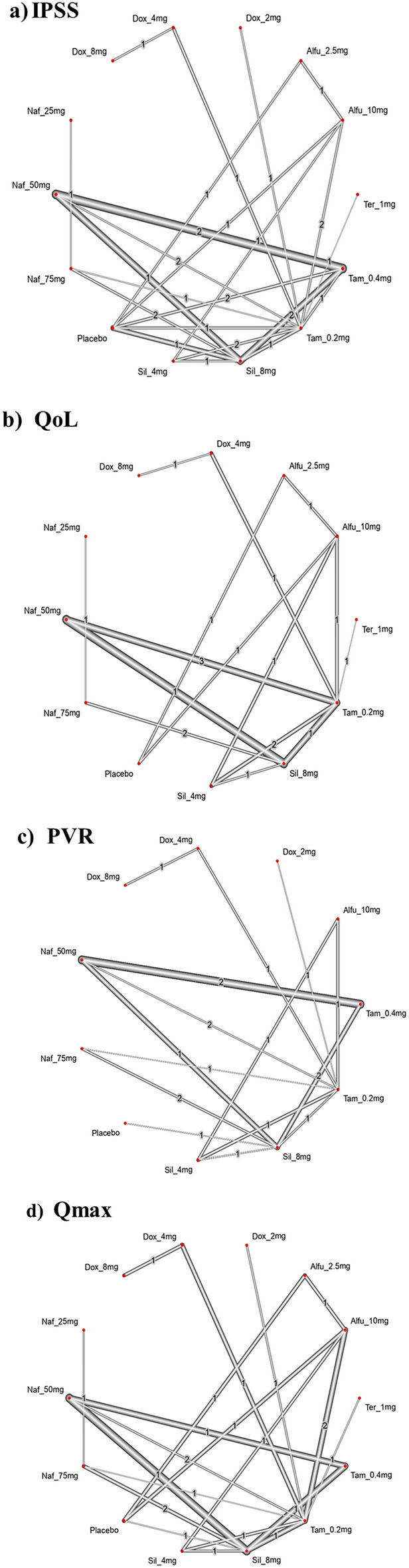
Network plot comparing individual α-blockers on international prostate symptom score (IPSS), quality of life (QoL), post-void residual volume (PVR) and maximum flow rate (Q max). The width of the edge is proportional to the number of trials comparing the two drugs, and the node represents the type of treatment. Tam = tamsulosin, Alfu = alfuzosin, Naf = naftopidil, Tera = terazosin, Dox = doxazosin, Sil = silodosin.

The NMA on IPSS included 22 RCTs with 6 interventions across 13 dose categories and 3271 participants (Fig. 2a). The base-case estimates of the efficacy of α-blockers regimens on reducing IPSS are listed in Table 2. Twenty-three comparisons estimated the treatment effect derived from direct evidence, 86 comparisons with indirect evidence and 18 comparisons with mixed evidence. Compared to the placebo, the NMA results found that three drugs had a significant effect on the reduction in IPSS, such as tamsulosin 0.4 mg (SMD: − 6.10; 95% CI: [− 8.74; − 3.47]), followed by naftopidil 50 mg (SMD: − 5.09; 95% CI: [− 8.29; − 1.89]) and silodosin 8 mg (SMD: − 3.63; 95% CI: [− 6.31; − 0.95]) (Fig. 3a). The relative effectiveness was depicted using the league table (Table S1), all included α-blockers significantly reduce the IPSS compared to the placebo. Based on the p-score the highest-ranked treatment was tamsulosin 0.4 mg (0.89) and the lowest-ranked treatment was doxazosin 2 mg (0.22) (Table 3). Furthermore, the Q test of consistency showed substantial heterogeneity for this comparison (I^2^, 85.5%) (Appendix S1).

**Table 3:** Ranking probability based on p-score for IPSS reduction.

Drug	Abbreviation	P score	Rank
Tamsulosin 0.4 mg	Tam_0.4mg	0.89	1
Naftopidil 50 mg	Naf_50mg	0.77	2
Terazosin 1 mg	Ter_1mg	0.72	3
Doxazosin 4 mg	Dox_4mg	0.67	4
Silodosin 4 mg	Sil_4mg	0.61	5
Silodosin 8 mg	Sil_8mg	0.57	6
Doxazosin 8 mg	Dox_8mg	0.49	7
Naftopidil 25 mg	Naf_25mg	0.46	8
Tamsulosin 0.2 mg	Tam_0.2mg	0.44	9
Naftopidil 75 mg	Naf_75mg	0.38	10
Alfuzosin 2.5 mg	Alfu_2.5mg	0.36	11
Alfuzosin 10 mg	Alfu_10mg	0.31	12
Doxazosin 2 mg	Dox_2mg	0.22	13
Placebo	Placebo	0.10	14

The higher scores reflected a higher probability of being the most effective treatment.

#### Quality of life (QoL)

13 RCTs including 6 interventions in 12 dose categories with 2,783 participants contributed to the comparison of the improvement in QoL (Fig. 2b). Fourteen comparisons estimated the treatment effect derived from direct evidence, 58 comparisons with indirect evidence and 7 comparisons with mixed evidence. Compared to the placebo, none of the comparison reached statistical significance in improving QoL (Fig. 3b). Doxazosin 8 mg has the highest probability of improving QoL, although the results were imprecise (Table S3). According to the pairwise comparisons, doxazosin 8 mg (− 1.35 [− 4.68; 1.98]) improves QoL compared to placebo (Table S2). Additionally, the Q consistency test showed a substantial heterogeneity for this evaluation (I^2^, 83.04%) (Appendix S1).

#### Post-void residual volume (PVR)

15 RCTs including 6 interventions in 10 dose categories with 2,761 participants contributed to the comparison of the reduction in PVR (Fig. 2c). Fifteen comparisons estimated the treatment effect derived from direct evidence, 51 comparisons with indirect evidence and 11 comparisons with mixed evidence. Compared to the placebo, none of the comparisons showed statistical significance in reducing PVR (Fig. 3c). Tamsulosin 0.4 mg and naftopidil 50 mg had the highest probability of improving PVR, with a p-score of 0.89, however, the results were imprecise (Table S5). According to the pairwise comparisons, tamsulosin 0.4 mg (− 15.99 [− 3.15; 35.12]) reduces the PVR compared to placebo; followed by naftopidil 50 mg (− 15.88 [− 34.73; 2.97]), doxazosin 2 mg (− 12.44[− 36.96; 12.07]) and doxazosin 4 mg (− 6.34 [− 28.27; 15.58]) (Table S4). Additionally, the Q consistency test showed a no heterogeneity for this evaluation (I^2^, 0%) (Appendix S1).

#### Maximum urinary flow rate (Qmax)

16 RCTs including 6 interventions in 13 dose categories with 3,114 participants contributed to the comparison of the improvement in Qmax (Fig. 2d). Twenty comparisons estimated the treatment effect derived from direct evidence, 60 comparisons with indirect evidence and 15 comparisons with mixed evidence. Compared to the placebo, none of the comparisons showed statistical significance in improving Qmax (Fig. 3d). Tamsulosin 0.4 mg has the highest probability of improving Qmax, with a p-score of 0.75 (Table S7). According to the pairwise comparisons, tamsulosin 0.4 mg (− 4.30 [− 9.37; 0.76]) reduces the Qmax compared to placebo; followed by terazosin 1 mg (− 3.99 [− 9.86; 1.89]), doxazosin 4 mg (− 3.39 [− 2.08; 8.86]) and naftopidil 75 mg (− 3.53 [− 3.03; 10.09]) (Table S6). Moreover, the Q consistency test showed a considerable heterogeneity for this evaluation (I^2^, 65.87%) (Appendix S1).

### Safety outcomes

A total of 297 AEs was reported among the α-blockers (events/participants = 297/3009), silodosin (190/739) dominated with a notable number of AEs followed by tamsulosin (32/966), doxazosin (27/313), naftopidil (25/544), alfuzosin (20/416) and terazosin (3/31). The most prominent AEs included ejaculation dysfunction, dizziness and hypotension. The AEs associated with α-blockers have been listed in Table S8.

### Evaluation of evidence quality

The degree of certainty of evidence for each outcome has been depicted in Figure S6, S7, S8, S9. About half of the comparison are moderate to low level confidence rating for IPSS vs placebo. Despite this, it was low for all other comparisons owing to imprecision and incoherence. However, the results of local and global approaches for IPSS showed inconsistent while all the other outcomes were found consistent. The quality scoring of the included studies is illustrated in the Figure S5. Furthermore, visual inspection of the comparison-adjusted funnel plots found the evidence of small-study effects for all outcomes (asymmetrical funnel plot) which indicates presence of potential publication bias (Figs. S1, S2, S3, S4).

## Discussion

Although there are several therapeutical options for BPH presently, pharmacological therapy has become standard care and is widely recommended by clinical guidelines^7^. American Urological Association (AUA) and Canadian Urological Association (CUA) guidelines recommend α- blockers as the first-line drug for BPH^45,46^. Despite their rapid onset of action, efficacy and modest frequency and intensity of adverse effects, α-blockers are considered as an excellent choice of therapy for BPH associated LUTS. The underlying mechanism of α-blockers is to inhibit the effect of norepinephrine produced endogenously on smooth muscle cells of the prostate; thereby reducing prostatic tone and consequently, urethral obstruction^47,48^. Several α-blockers have been approved by the FDA for the treatment of BPH, including terazosin, alfuzosin, doxazosin, tamsulosin and silodosin whereas naftopidil is only approved in Japan^49–51^.

Various clinical trials have been performed to investigate the effectiveness of α- blockers for BPH, however direct comparisons among many drugs are still lacking^12–14^. At present, none of the NMA have extensively evaluated the efficacy of these agents within the class in terms of the majority of outcomes (IPSS, QoL, PVR, Qmax) as well as TEAEs. This NMA focused on 22 RCTs, which included 3271 patients randomly assigned to 6 kinds of α-blockers or placebo with 12 dose categories. Our study revealed that among all the α-blocker monotherapy, tamsulosin 0.4 mg is more effective in improving the IPSS, PVR and Qmax, compared to a placebo, as well as the highest-ranked treatment option for these outcomes based on the rank test. Silodosin is considered to be having the highest selectivity for α1A adrenoreceptors in comparison to other α-blockers. In-vitro studies have shown that the affinity of silodosin and tamsulosin for α1A adrenoreceptors over α1B adrenoreceptors was 580-fold and 55-fold respectively. Based on this several clinical trials have also shown that silodosin has greater or comparable efficacy to tamsulosin. However, our NMA contradicts the above observations^13,44^ and it clearly suggests the so called highly selective α-blocker, silodosin is not superior to tamsulosin in terms of clinical outcomes. This will help urologists in better counselling the BPH patients with regard to efficacy of different α-blockers. All the included α-blockers in our study showed a promising effect in reducing the IPSS. On the other hand, α-blockers did not significantly improve QoL, although they showed numerically better results. Even though, the pairwise comparison has shown that doxazosin 8 mg considerably improves QoL more than other α-blockers and is the highest-ranked treatment choice in the rank test.

Most guidelines routinely recommended using a symptom questionnaire to evaluate the patient's symptoms. IPSS, is the most ordinarily preferred scoring system, which is based on the American Urological Association Symptom Index (AUA-SI)^15,16^. It comprises eight questions, seven of which explore urinary symptoms and one on the overall quality of life^52^. All of the included α-blockers significantly reduced IPSS within the first 2 weeks of treatment. Controlled studies suggest that α-blockers often lower the IPSS by 30–40%^47^. In addition to their remarkable efficacy, α-blockers are the least expensive and well-tolerated of the drugs used to treat LUTS^16,53^.

The included studies validated the overall safety profile, with the proportion of AEs ranging mild to moderate. The most commonly reported AEs were ejaculation disorder, dizziness, diarrhoea, nasal congestion, drowsiness and postural hypotension. Moreover, for each of the aforementioned α-blockers, dizziness was reported. Wang et al. observed similar findings, stating that the most commonly reported AEs with α-blockers were ejaculation disorders, nasopharyngitis, and vasodilation effects such as asthenia, dizziness, headache and hypotension^15^. As compared to other α-blockers, silodosin elicits a notable number of AEs followed by tamsulosin and doxazosin and the most predominant adverse effects were ejaculation dysfunction, dizziness, and hypotension. In addition to corroborating our findings, investigations on those most recent drug treatments for LUTS also concurred that silodosin have a higher AE profile than the other therapies, exhibiting with a higher rate of ejaculation dysfunction^54,55^. However, α-blockers monotherapies are generally safe with relatively few AEs.

This is the first robust network meta-analysis purely focused on α-blockers, considering the majority of outcomes (IPSS, QoL, PVR, Qmax) along with TEAEs. In 2015, Yuan et al. performed a NMA of RCTs for evaluating the comparative effectiveness of monodrug therapies in BPH^16^. However, outcomes such as PVR and QoL were not considered. Moreover, numerous studies were published after 2015 (36.4%), resulting in the up-to-date comparison of interventions. Studies conducted by Lepor et al. found that when comparing different α-blockers, it is imperative to consider that efficacy and safety are dose-dependent. As a result, observed differences in efficacy and toxicity may be related to diverse levels of α1-blockade achieved rather than inherent pharmacological advantages of the specific drug^8^. We compared α-blockers in a dose-dependent way to benefit the comparative efficacy and safety at different dose levels. Furthermore, the selected studies had similar study designs, selection criteria, and patient characteristics with few exception (duration of treatment) thus, supporting exchangeability. Exchangeability across the trials were conceptually considered and the NMA findings were interpreted accordingly. These factors enhance the credibility of the comparisons generated. Besides, the overall quality of the studies selected was found satisfactory.

Although we performed a comprehensive systematic review and NMA of α-blockers, there are still constraints to consider when interpreting the findings. This review focused on four outcomes, but there were limited data available for QoL, PVR, and Qmax as compared to IPSS. The majority of comparisons for outcomes such as PVR and QoL exhibited low certainty of evidence with the CINeMA framework, predominantly implying the risk of bias from the open-label trials and imprecision owing to a relatively small number of trials. Secondly, α-blockers can minimize both storage and voiding LUTS, however, prostate size has no effect in short-term studies (≤ 1 year)^56,57^. The conventional clinical treatment for larger prostate size requires a prolonged treatment period^15^. Ipso facto, the limited duration in the included RCTs (50% of studies were ≤ 8 weeks and 45% ≤ 12 weeks) impede the estimation of long-term effects of α-blockers. Furthermore, this study assessed the efficacy and safety of six different kinds of α-blockers, including five drugs approved by the US FDA (terazosin, alfuzosin, doxazosin, silodosin, and tamsulosin) for BPH while naftopidil is only approved in Japan. As a result, the findings of naftopidil cannot be generalised. Furthermore, the majority of studies were conducted in Asian countries, which could impact the broader applicability of the results. The safety of different kinds of α-blockers was not evaluated using NMA due to a lack of information and the diversity of TEAEs. When interpreting the outcomes of this study, it is imperative to consider the imprecision, heterogeneity and incoherence inherent in the effect estimates.

## Conclusion

All the included α-blockers showed reduction in IPSS whereas tamsulosin 0.4 mg outperforms the other α-blocker monotherapies in terms of improving IPSS, PVR, and Qmax. Moreover, larger sample sizes along with longer-term studies are required to refine our estimates of IPSS, QoL, PVR, and Qmax among α-blocker users. Silodosin elicits a notable number of AEs however, dizziness was a common AE observed for all α-blockers. Despite the advancing volume of evidence on the α-blocker, there remains a paucity of evidence demonstrating comparative safety in terms of serious and unexpected outcomes. Even though results provide a pragmatic evaluation of six different types of α-blockers that can aid in treatment decisions, direct head-to-head comparisons are required to validate these findings.

### Supplementary Information

Supplementary Information.

## Data Availability

The datasets gathered in the present study are considered for sharing upon reasonable requests to the corresponding author.
