# Author Correction: Comparative efficacy and safety of alpha-blockers as monotherapy for benign prostatic hyperplasia: a systematic review and network meta-analysis

**DOI:** 10.1038/s41598-024-63406-z

**Published:** 2024-06-05

**Authors:** Beema T Yoosuf, Abhilash Kumar Panda, Muhammed Favas KT, Saroj Kundan Bharti, Sudheer Kumar Devana, Dipika Bansal

**Affiliations:** 1 Department of Pharmacy Practice, National Institute of Pharmaceutical Education and Research (NIPER), S.A.S. Nagar, Mohali, Punjab India; 2 Department of Urology, Postgraduate Institute of Medical Education and Research, Chandigarh, India

Correction to: *Scientific Reports* 10.1038/s41598-024-61977-5, published online 15 May 2024

The original version of this Article contained errors in the legends of Figure 2 and Figure 3, which were inadvertently switched.

The Legend of Figure 2

“Network plot comparing individual α-blockers on international prostate symptom score (IPSS), quality of life (QoL), post-void residual volume (PVR) and maximum flow rate (Q max). The width of the edge is proportional to the number of trials comparing the two drugs, and the node represents the type of treatment. Tam = tamsulosin, Alfu = alfuzosin, Naf = naftopidil, Tera = terazosin, Dox = doxazosin, Sil = silodosin.”

now reads:

“Forest plot of interventions as measured by the international prostate symptom score (IPSS), quality of life (QoL), post-void residual volume (PVR) and maximum flow rate (Q max). Tam = tamsulosin, Alfu = alfuzosin, Naf = naftopidil, Tera = terazosin, Dox = doxazosin, Sil = silodosin.”

The Legend of Figure 3

“Forest plot of interventions as measured by the international prostate symptom score (IPSS), quality of life (QoL), post-void residual volume (PVR) and maximum flow rate (Q max). Tam = tamsulosin, Alfu = alfuzosin, Naf = naftopidil, Tera = terazosin, Dox = doxazosin, Sil = silodosin.”

now reads:

“Network plot comparing individual α-blockers on international prostate symptom score (IPSS), quality of life (QoL), post-void residual volume (PVR) and maximum flow rate (Q max). The width of the edge is proportional to the number of trials comparing the two drugs, and the node represents the type of treatment. Tam = tamsulosin, Alfu = alfuzosin, Naf = naftopidil, Tera = terazosin, Dox = doxazosin, Sil = silodosin.”

The original version of this Article has been corrected.

