# Supplementary material for: Comparative efficacy and safety of alpha-blockers as monotherapy for benign prostatic hyperplasia: a systematic review and network meta-analysis
Source: Sci Rep. 2024 May 15;14:11116. doi: 10.1038/s41598-024-61977-5 (PMC11096304; doi:10.1038/s41598-024-61977-5)

Comparative Efficacy and Safety of Alpha-Blockers as Single Agent for Benign Prostatic Hyperplasia: A Systematic Review and Network Meta-Analysis.

Supplementary Material

**Table S1:** League table for the relative effectiveness of interventions based on IPSS

**Figure S1:** Comparison-adjusted funnel plots of IPSS

**Table S2:** League table for the relative effectiveness of interventions based on QoL

**Table S3:** Ranking probability based on p-score for improvement in QoL

**Figure S2:** Comparison-adjusted funnel plots of QoL

**Table S4:** League table for the relative effectiveness of interventions based on PVR

**Table S5:** Ranking probability based on p-score for the reduction in PVR.

**Figure S3:** Comparison-adjusted funnel plots for PVR

**Table S6:** League table for the relative effectiveness of interventions based on Qmax

**Table S7:** Ranking probability based on p-score for improvement in Qmax

**Figure S4:** Comparison-adjusted funnel plots for Qmax

**Table S8:** Treatment-emergent adverse events (TEAEs)

**Figure S5:** Risk of bias of included studies

**Figure S6:** GRADE rating for comparisons of IPSS.

**Figure S7:** GRADE rating for comparisons of QoL

**Figure S8:** GRADE rating for comparisons of QoL

**Figure S9:** GRADE rating for comparisons of Qmax

**Table S9:** Search strategy developed in various databases

**Table S10:** List of studies excluded at full-text screening stage, with brief reasons.

**Appendix S1:** Details on Inconsistency

**Table S1: League table for the relative effectiveness of interventions based on IPSS**

| **Alfu_10mg** |  |  |  |  |  |  |  |  |  |  |  |  |  |
| --- | --- | --- | --- | --- | --- | --- | --- | --- | --- | --- | --- | --- | --- |
| 0.13  [ -4.09; 4.36] | **Alfu_2.5mg** |  |  |  |  |  |  |  |  |  |  |  |  |
| -1.25  [ -6.72; 4.22] | -1.38  [ -7.90; 5.14] | **Dox_2mg** |  |  |  |  |  |  |  |  |  |  |  |
| 2.65  [ -2.63; 7.94] | 2.52  [ -3.85; 8.89] | 3.90  [ -2.56; 10.36] | **Dox_4mg** |  |  |  |  |  |  |  |  |  |  |
| 1.25  [ -5.77; 8.27] | 1.12  [ -6.75; 8.99] | 2.50  [ -5.44; 10.44] | -1.40  [ -6.02; 3.22] | **Dox_8mg** |  |  |  |  |  |  |  |  |  |
| 0.90  [ -5.44; 7.24] | 0.77  [ -6.43; 7.97] | 2.15  [ -5.32; 9.62] | -1.75  [ -9.09; 5.59] | -0.35  [ -9.02; 8.32] | **Naf_25mg** |  |  |  |  |  |  |  |  |
| 3.17  [ -0.47; 6.81] | 3.04  [ -1.94; 8.02] | 4.42  [ -0.94; 9.78] | 0.52  [ -4.65; 5.69] | 1.92  [ -5.02; 8.86] | 2.27  [ -3.87; 8.41] | **Naf_50mg** |  |  |  |  |  |  |  |
| 0.50  [ -3.60; 4.60] | 0.37  [ -4.97; 5.71] | 1.75  [ -3.94; 7.45] | -2.15  [ -7.67; 3.37] | -0.75  [ -7.95; 6.45] | -0.40  [ -5.23; 4.43] | -2.67  [ -6.46; 1.12] | **Naf_75mg** |  |  |  |  |  |  |
| -1.92  [ -5.08; 1.24] | -2.06  [ -6.31; 2.20] | -0.67  [ -6.07; 4.73] | -4.57  [ -9.79; 0.64] | -3.17  [-10.14; 3.80] | -2.82  [ -8.95; 3.30] | -5.09  [ -8.29; -1.89] | -2.42  [ -6.18; 1.34] | **Placebo** |  |  |  |  |  |
| 2.01  [ -1.42; 5.45] | 1.88  [ -3.13; 6.89] | 3.26  [ -2.21; 8.74] | -0.64  [ -5.93; 4.66] | 0.76  [ -6.26; 7.79] | 1.11  [ -5.15; 7.38] | -1.16  [ -4.79; 2.47] | 1.51  [ -2.48; 5.50] | -3.94  [ -7.47; -0.40] | **Sil_4mg** |  |  |  |  |
| 1.71  [ -1.57; 4.99] | 1.57  [ -3.12; 6.27] | 2.96  [ -2.25; 8.17] | -0.94  [ -5.96; 4.08] | 0.46  [ -6.37; 7.28] | 0.81  [ -4.84; 6.45] | -1.46  [ -4.24; 1.31] | 1.21  [ -1.72; 4.13] | -3.63  [- 6.31; -0.95] | -0.31  [ -3.37; 2.76] | **Sil_8mg** |  |  |  |
| 0.95  [ -1.89; 3.79] | 0.82  [ -3.73; 5.37] | 2.20  [ -2.47; 6.87] | -1.70  [ -6.16; 2.76] | -0.30  [ -6.72; 6.12] | 0.05  [ -5.78; 5.88] | -2.22  [ -4.85; 0.41] | 0.45  [ -2.81; 3.71] | -2.87  [ 0.16; 5.58] | -1.06  [ -3.92; 1.80] | -0.76  [ -3.07; 1.55] | **Tam_0.2mg** |  |  |
| 4.18  [ 0.76; 7.61] | 4.05  [ -0.68; 8.78] | 5.43  [ 0.11; 10.75] | 1.53  [ -3.60; 6.66] | 2.93  [ -3.97; 9.84] | 3.28  [ -2.74; 9.30] | 1.01  [ -1.56; 3.59] | 3.68  [ 0.09; 7.27] | -6.10  [ -3.47; -8.74] | 2.17  [ -1.30; 5.63] | 2.47  [ 0.08; 4.87] | 3.23  [ 0.69; 5.78] | **Tam_0.4mg** |  |
| 3.35  [ -2.92; 9.62] | 3.22  [ -3.99; 10.42] | 4.60  [ -2.68; 11.88] | 0.70  [ -6.45; 7.85] | 2.10  [ -6.41; 10.61] | 2.45  [ -5.62; 10.52] | 0.18  [ -6.00; 6.35] | 2.85  [ -3.62; 9.32] | -5.27  [ -0.94; 11.48] | 1.34  [ -4.94; 7.61] | 1.64  [ -4.41; 7.69] | 2.40  [ -3.19; 7.99] | -0.83  [ -6.97; 5.31] | **Ter_1mg** |

Tam = tamsulosin, Alfu = alfuzosin, Naf = naftopidil, Tera = terazosin, Dox = doxazosin, Sil = silodosin.

**Figure S1: Comparison-adjusted funnel plots of IPSS.**

***
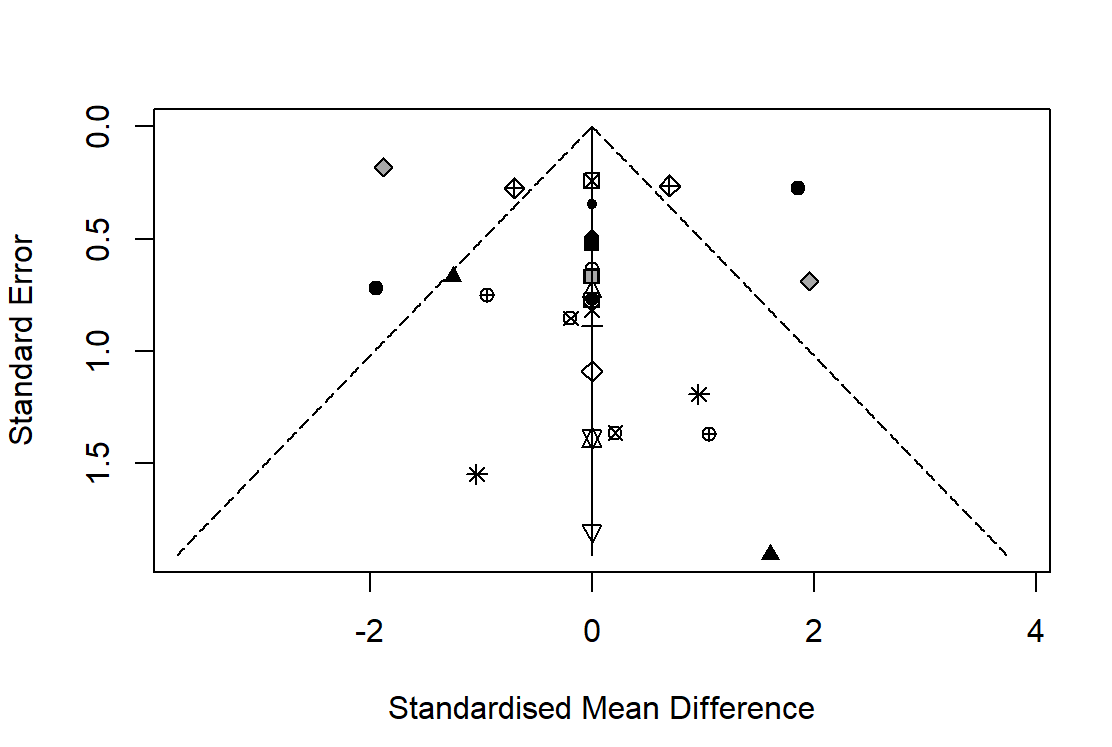
***

*Funnel plots have been generated solely for outcomes with 10 studies. (comparisons of treatments using a study-specific common comparator)

**Table S2: League table for the relative effectiveness of interventions based on QoL**

| **Alfu_10mg** |  |  |  |  |  |  |  |  |  |  |  |
| --- | --- | --- | --- | --- | --- | --- | --- | --- | --- | --- | --- |
| 0.00  [-1.69; 1.69] | **Alfu_2.5 mg** |  |  |  |  |  |  |  |  |  |  |
| 0.35  [-1.95; 2.64] | 0.35  [-2.50; 3.20] | **Dox_4mg** |  |  |  |  |  |  |  |  |  |
| 0.95  [-1.92; 3.82] | 0.95  [-2.38; 4.28] | 0.60  [-1.12; 2.32] | **Dox_8mg** |  |  |  |  |  |  |  |  |
| -0.88  [-3.69; 1.93] | -0.88  [-4.16; 2.40] | -1.23  [-4.20; 1.75] | -1.83  [-5.26; 1.61] | **Naf_25mg** |  |  |  |  |  |  |  |
| -0.13  [-1.95; 1.68] | -0.13  [-2.61; 2.35] | -0.48  [-2.43; 1.47] | -1.08  [-3.69; 1.52] | 0.74  [-1.77; 3.26] | **Naf_50mg** |  |  |  |  |  |  |
| -0.78  [-2.99; 1.44] | -0.78  [-3.57; 2.01] | -1.13  [-3.55; 1.29] | -1.73  [-4.70; 1.24] | 0.10  [-1.63; 1.83] | -0.64  [-2.47; 1.18] | **Naf_75mg** |  |  |  |  |  |
| -0.40  [-2.10; 1.30] | -0.40  [-2.09; 1.29] | -0.75  [-3.60; 2.10] | -1.35  [-4.68; 1.98] | 0.48  [-2.80; 3.76] | -0.27  [-2.75; 2.22] | 0.38  [-2.41; 3.17] | **Placebo** |  |  |  |  |
| -0.12  [-1.68; 1.44] | -0.12  [-2.42; 2.18] | -0.47  [-2.48; 1.54] | -1.07  [-3.71; 1.58] | 0.76  [-1.72; 3.24] | 0.02  [-1.38; 1.41] | 0.66  [-1.11; 2.43] | -0.28  [-2.02; 2.58] | **Sil_4mg** |  |  |  |
| -0.18  [-2.04; 1.67] | -0.18  [-2.69; 2.33] | -0.53  [-2.62; 1.56] | -1.13  [-3.84; 1.58] | 0.70  [-1.42; 2.81] | -0.05  [-1.41; 1.31] | 0.60  [-0.62; 1.81] | -0.22  [-2.29; 2.73] | -0.06  [-1.36; 1.23] | **Sil_8mg** |  |  |
| -0.05  [-1.61; 1.51] | -0.05  [-2.35; 2.25] | -0.40  [-2.08; 1.28] | -1.00  [-3.41; 1.41] | 0.83  [-1.63; 3.28] | 0.08  [-0.91; 1.07] | 0.73  [-1.01; 2.47] | -0.35  [-1.95; 2.65] | 0.07  [-1.03; 1.16] | 0.13  [-1.11; 1.37] | **Tam_0.2mg** |  |
| 0.25  [-2.15; 2.65] | 0.25  [-2.69; 3.18] | -0.10  [-2.58; 2.38] | -0.70  [-3.72; 2.32] | 1.13  [-1.93; 4.18] | 0.38  [-1.69; 2.46] | 1.03  [-1.49; 3.55] | -0.65  [-2.29; 3.59] | 0.37  [-1.76; 2.50] | 0.43  [-1.78; 2.64] | 0.30  [-1.52; 2.12] | **Ter_1mg** |

Tam = tamsulosin, Alfu = alfuzosin, Naf = naftopidil, Tera = terazosin, Dox = doxazosin, Sil = silodosin.

**Table S3: Ranking probability based on p-score for improvement in QoL**

| **Drug** | **Abbreviation** | **P score** | **Rank** |
| --- | --- | --- | --- |
| Doxazosin 8mg | Dox_8mg | 0.78 | 1 |
| Doxazosin 4mg | Dox_4mg | 0.64 | 2 |
| Terazosin 1mg | Ter_1mg | 0.61 | 3 |
| Alfuzosin 10 mg | Alfu_10mg | 0.54 | 4 |
| Alfuzosin 2.5 mg | Alfu_2.5 mg | 0.53 | 5 |
| Tamsulosin 0.2mg | Tam_0.2mg | 0.52 | 6 |
| Silodosin 4mg | Sil_4mg | 0.49 | 7 |
| Naftopidil 50mg | Naf_50mg | 0.48 | 8 |
| Silodosin 8mg | Sil_8mg | 0.47 | 9 |
| Placebo | Placebo | 0.40 | 10 |
| Naftopidil 25mg | Naf_25mg | 0.28 | 11 |
| Naftopidil 75mg | Naf_75mg | 0.26 | 12 |

The higher scores reflected a higher probability of being the most

effective treatment.

**Figure S2: Comparison-adjusted funnel plots of QoL**


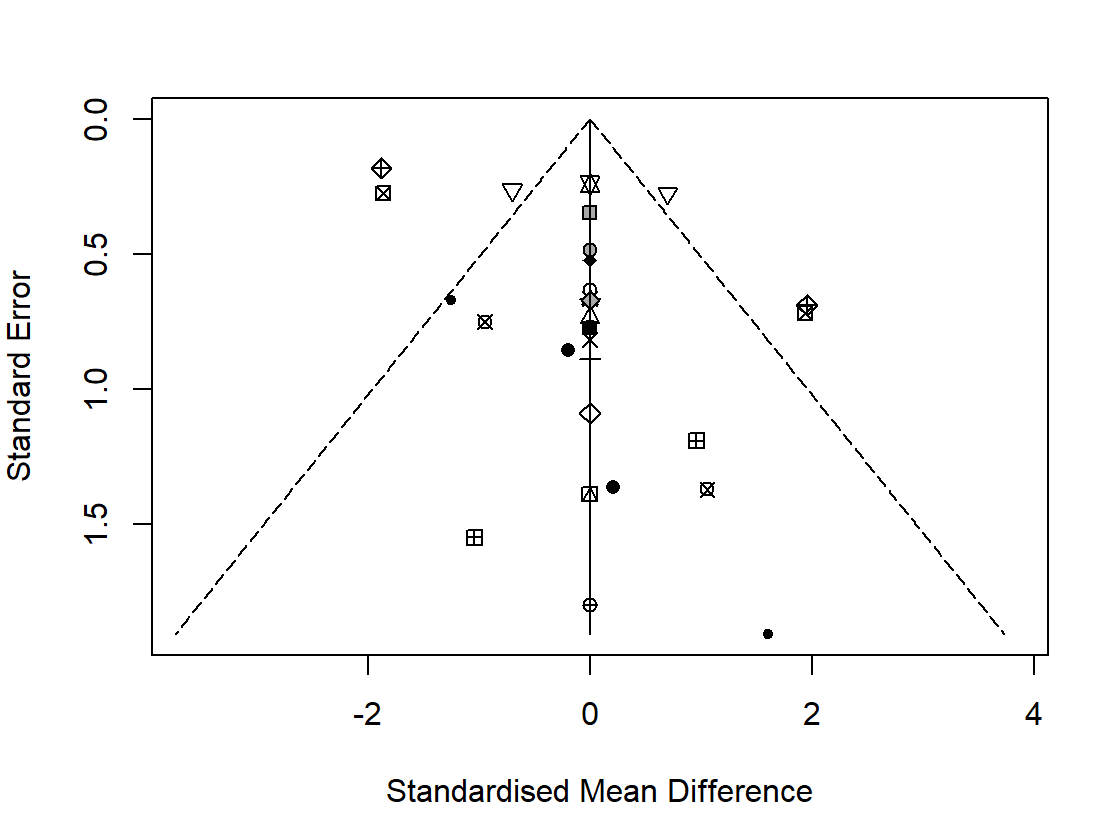


*Funnel plots have been generated solely for outcomes with 10 studies.

(comparisons of treatments using a study-specific common comparator)

**Table S4: League table for the relative effectiveness of interventions based on PVR**

| **Alfu_10mg** |  |  |  |  |  |  |  |  |  |  |
| --- | --- | --- | --- | --- | --- | --- | --- | --- | --- | --- |
| 10.86  [ -8.12; 29.85] | **Dox_2mg** |  |  |  |  |  |  |  |  |  |
| 4.76  [-10.73; 20.26] | -6.10  [-25.55; 13.35] | **Dox_4mg** |  |  |  |  |  |  |  |  |
| -2.44  [-21.94; 17.06] | -13.30  [-36.07; 9.47] | -7.20  [-19.04; 4.64] | **Dox_8mg** |  |  |  |  |  |  |  |
| 14.30  [ 0.90; 27.70] | 3.44  [-14.74; 21.62] | 9.54  [ -4.96; 24.03] | 16.74  [ -1.97; 35.45] | **Naf_50mg** |  |  |  |  |  |  |
| -3.32  [-17.81; 11.18] | -14.18  [-33.25; 4.89] | -8.08  [-23.68; 7.52] | -0.88  [-20.46; 18.70] | -17.62  [-29.31; -5.93] | **Naf_75mg** |  |  |  |  |  |
| -1.58  [-22.62; 19.46] | -12.44  [-36.96; 12.07] | -6.34  [-28.27;15.58] | 0.86  [-24.06; 25.77] | -15.88  [-34.73; 2.97] | 1.74  [-17.59; 21.06] | **Placebo** |  |  |  |  |
| -0.94  [-11.52; 9.64] | -11.80  [-30.04; 6.44] | -5.70  [-20.27; 8.87] | 1.50  [-17.28; 20.27] | -15.24  [-27.01; -3.47] | 2.38  [-10.54; 15.29] | 0.64  [-19.21; 20.50] | **Sil_4mg** |  |  |  |
| 5.42  [ -6.92; 17.75] | -5.44  [-23.06; 12.18] | 0.66  [-13.13; 14.45] | 7.86  [-10.32; 26.03] | -8.88  [-16.93; -0.84] | 8.74  [ -0.36; 17.83] | 7.00  [-10.05; 24.05] | 6.36  [-3.83; 16.54] | **Sil_8mg** |  |  |
| 2.36  [ -8.18; 12.91] | -8.50  [-24.29; 7.29] | -2.40  [-13.75; 8.95] | 4.80  [-11.60; 21.20] | -11.94  [-20.95; -2.93] | 5.68  [ -5.02; 16.37] | -3.94  [-14.81; 22.70] | 3.30  [ -5.83; 12.44] | -3.06  [-10.88; 4.77] | **Tam_0.2mg** |  |
| 14.40  [ 0.22; 28.59] | 3.54  [-15.28; 22.37] | 9.64  [ -5.66; 24.94] | 16.84  [ -2.50; 36.19] | 0.10  [ -6.86; 7.07] | 17.72  [ 5.44; 30.00] | -15.99  [ -3.15; 35.12] | 15.34  [ 2.77; 27.92] | 8.99  [ 0.29; 17.68] | 12.04  [1.79; 22.29] | **Tam_0.4mg** |

Tam = tamsulosin, Alfu = alfuzosin, Naf = naftopidil, Tera = terazosin, Dox = doxazosin, Sil = silodosin.

**Table S5: Ranking probability based on p-score for reduction in PVR.**

| **Drug** | **Abbreviation** | **P score** | **Rank** |
| --- | --- | --- | --- |
| Naftopidil 50mg | Naf_50mg | 0.89 | 1 |
| Tamsulosin 0.4mg | Tam_0.4mg | 0.89 | 2 |
| Doxazosin 2mg | Dox_2mg | 0.74 | 3 |
| Silodosin 8mg | Sil_8mg | 0.59 | 4 |
| Doxazosin 4mg | Dox_4mg | 0.55 | 5 |
| Tamsulosin 0.4mg | Tam_0.2mg | 0.44 | 6 |
| Alfuzosin 10mg | Alfu_10mg | 0.34 | 7 |
| Placebo | Placebo | 0.31 | 8 |
| Silodosin 4mg | Sil_4mg | 0.28 | 9 |
| Doxazosin 8mg | Dox_8mg | 0.27 | 10 |
| Naftopidil 75mg | Naf_75mg | 0.20 | 11 |

The higher score reflected a higher probability of being the most

effective treatment.

**Figure S3: Comparison-adjusted funnel plots for PVR**


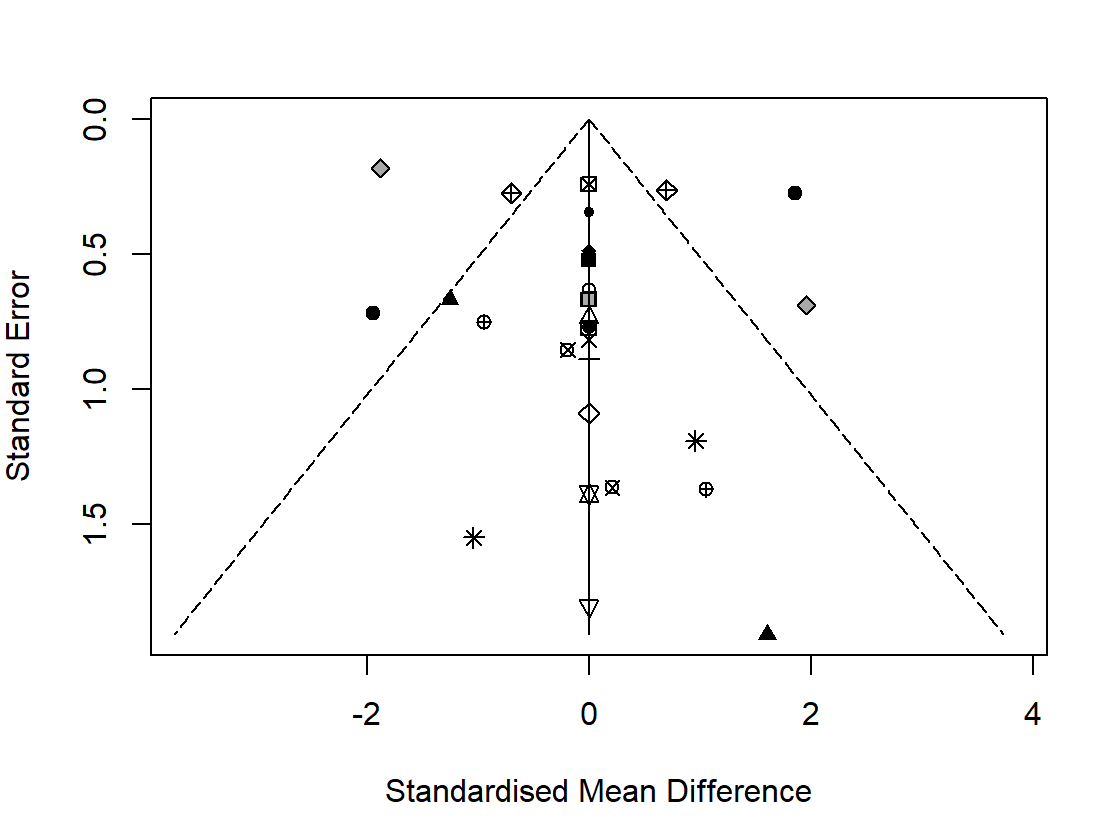


*Funnel plots have been generated solely for outcomes with 10 studies.

(comparisons of treatments using a study-specific common comparator)

**Table S6: League table for the relative effectiveness of interventions based on Qmax**

| **Alfu_10mg** |  |  |  |  |  |  |  |  |  |  |  |  |  |
| --- | --- | --- | --- | --- | --- | --- | --- | --- | --- | --- | --- | --- | --- |
| 0.33  [ -3.55; 4.22] | **Alfu_2.5 mg** |  |  |  |  |  |  |  |  |  |  |  |  |
| 0.67  [ -4.36; 5.70] | 0.34  [ -5.85; 6.52] | **Dox_2mg** |  |  |  |  |  |  |  |  |  |  |  |
| -1.23  [ -6.02; 3.57] | -1.56  [ -7.56; 4.43] | -1.90  [ -7.76; 3.96] | **Dox_4mg** |  |  |  |  |  |  |  |  |  |  |
| -0.93  [ -7.21; 5.36] | -1.26  [ -8.51; 5.98] | -1.60  [ -8.74; 5.54] | 0.30  [ -3.76; 4.36] | **Dox_8mg** |  |  |  |  |  |  |  |  |  |
| -0.24  [ -3.84; 3.36] | -0.58  [ -5.60; 4.45] | -0.91  [ -5.97; 4.14] | 0.99  [ -3.84; 5.81] | 0.69  [ -5.62; 6.99] | **Naf_50mg** |  |  |  |  |  |  |  |  |
| -0.37  [ -4.22; 3.48] | -0.70  [ -5.85; 4.45] | -1.04  [ -6.38; 4.30] | 0.86  [ -4.26; 5.98] | 0.56  [ -5.98; 7.09] | -0.13  [ -3.86; 3.61] | **Naf_75mg** |  |  |  |  |  |  |  |
| -1.37  [ -7.58; 4.84] | -1.70  [ -8.79; 5.39] | -2.04  [ -9.27; 5.19] | -0.14  [ -7.21; 6.93] | -0.44  [ -8.59; 7.71] | -1.13  [ -7.27; 5.01] | -1.00  [ -5.87; 3.87] | **Naf_75mg** |  |  |  |  |  |  |
| -2.16  [ -1.19; 5.52] | -1.83  [ -2.05; 5.70] | -1.49  [ -4.19; 7.17] | -3.39  [ -2.08; 8.86] | -3.09  [ -3.73; 9.90] | -2.40  [ -1.90; 6.71] | 2.53  [ -1.86; 6.92] | -3.53  [ -3.03; 10.09] | **Placebo** |  |  |  |  |  |
| -0.29  [ -3.60; 3.02] | -0.63  [ -5.51; 4.25] | -0.97  [ -6.23; 4.29] | 0.93  [ -4.10; 5.97] | 0.63  [ -5.84; 7.11] | -0.05  [ -3.83; 3.72] | 0.08  [ -3.83; 3.98] | 1.08  [ -5.17; 7.32] | -2.45  [ -6.67; 1.76] | **Sil_4mg** |  |  |  |  |
| -0.54  [ -3.64; 2.56] | -0.87  [ -5.44; 3.69] | -1.21  [ -6.12; 3.69] | 0.69  [ -3.98; 5.35] | 0.39  [ -5.80; 6.58] | -0.30  [ -3.19; 2.59] | -0.17  [ -2.88; 2.54] | 0.83  [ -4.75; 6.41] | -2.70  [ -6.33; 0.93] | -0.25  [ -3.30; 2.81] | **Sil_8mg** |  |  |  |
| -0.63  [ -3.26; 2.01] | -0.96  [ -5.42; 3.50] | -1.30  [ -5.58; 2.98] | 0.60  [ -3.41; 4.61] | 0.30  [ -5.41; 6.01] | -0.39  [ -3.08; 2.30] | -0.26  [ -3.44; 2.93] | 0.74  [ -5.08; 6.56] | -2.79  [ -6.52; 0.94] | -0.33  [ -3.39; 2.72] | -0.09  [ -2.48; 2.30] | **Tam_0.2mg** |  |  |
| -2.14  [ -6.73; 2.44] | -2.48  [ -8.21; 3.25] | -2.82  [ -8.70; 3.07] | -0.92  [ -6.60; 4.77] | -1.22  [ -8.20; 5.77] | -1.90  [ -5.64; 1.83] | -1.77  [ -6.29; 2.74] | -0.77  [ -7.42; 5.87] | -4.30  [ -9.37; 0.76] | -1.85  [ -6.49; 2.79] | -1.60  [ -5.34; 2.13] | -1.52  [ -5.55; 2.52] | **Tam_0.4mg** |  |
| -1.83  [ -7.08; 3.42] | -2.16  [ -8.53; 4.20] | -2.50  [ -8.74; 3.74] | -0.60  [ -6.65; 5.45] | -0.90  [ -8.19; 6.39] | -1.59  [ -6.86; 3.69] | -1.46  [ -7.01; 4.09] | -0.46  [ -7.84; 6.92] | -3.99  [ -9.86; 1.89] | -1.53  [ -7.01; 3.94] | -1.29  [ -6.42; 3.84] | -1.20  [ -5.74; 3.34] | 0.32  [ -5.76; 6.39] | **Ter_1mg** |

Tam = tamsulosin, Alfu = alfuzosin, Naf = naftopidil, Tera = terazosin, Dox = doxazosin, Sil = silodosin.

**Table S7: Ranking probability based on p-score for improvement in Qmax**

| **Drug** | **Abbreviation** | **P-score** | **Rank** |
| --- | --- | --- | --- |
| Tamsulosin 0.4mg | Tam_0.4mg | 0.75 | 1 |
| Terazosin 1mg | Tera_1mg | 0.68 | 2 |
| Doxazosin 4mg | Dox_4mg | 0.62 | 3 |
| Silodosin 8mg | Naf_75mg | 0.61 | 4 |
| Doxazosin 8mg | Dox_8mg | 0.56 | 5 |
| Tamsulosin 0.2mg | Tam_0.2mg | 0.54 | 6 |
| Silodosin 8mg | Sil_8mg | 0.52 | 7 |
| Naftopidil 75mg | Naf_75mg | 0.48 | 8 |
| Silodosin 4mg | Sil_4mg | 0.48 | 9 |
| Naftopidil 50mg | Naf_50mg | 0.46 | 10 |
| Alfuzosin 10 mg | Alfu_10mg | 0.42 | 11 |
| Alfuzosin 2.5mg | Alfu_2.5 mg | 0.40 | 12 |
| Doxazosin 2mg | Dox_2mg | 0.35 | 13 |
| Placebo | Placebo | 0.13 | 14 |

The higher score reflected a higher probability of being the most

effective treatment.

**Figure S4: Comparison-adjusted funnel plots for Qmax**


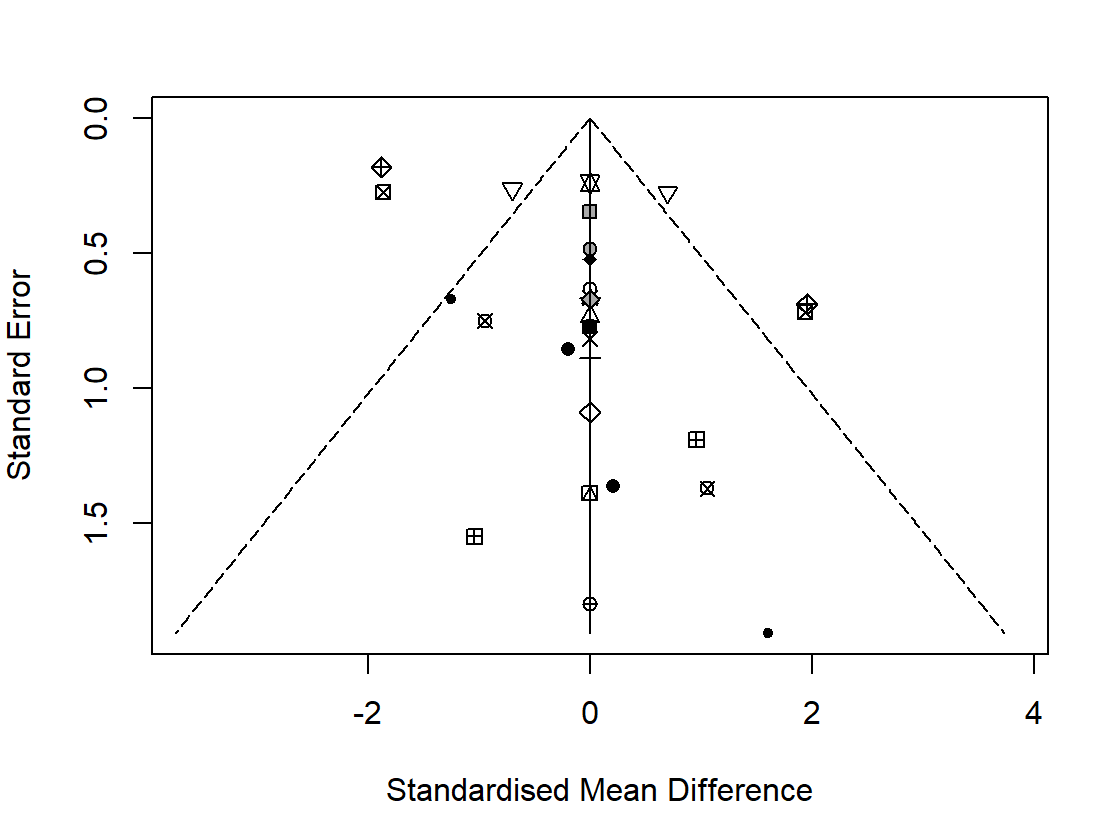


*Funnel plots have been generated solely for outcomes with 10 studies.

(comparisons of treatments using a study-specific common comparator)

**Table S8: Treatment-emergent adverse events (TEAEs)**

| **Adverse Event** | **Silodosin**  **(n=739)** | **Doxazosin**  **(n=313)** | **Tamsulosin**  **(n=966)** | **Naftopidil**  **(n=544)** | **Alfuzosin**  **(n=416)** | **Terazosin**  **(n=31)** | **Total**  **(n=3009)** |
| --- | --- | --- | --- | --- | --- | --- | --- |
| Ejaculation Disorder | 130 | 3 | 7 | 11 | NR | NR | **151** |
| Dizziness | 22 | 11 | 15 | 4 | 8 | 1 | **61** |
| Diarrhoea & Soft Stools | 11 | NR | NR | 2 | NR | NR | **13** |
| Nasal Congestion | 9 | NR | NR | NR | NR | NR | **9** |
| Drowsiness | NR | 8 | NR | NR | NR | NR | **8** |
| Postural Hypotension | NR | 1 | 3 | 4 | 1 | NR | **9** |
| Gastric Pain | NR | 2 | NR | NR | NR | NR | **2** |
| Palpitation | NR | 1 | NR | NR | NR | 1 | **2** |
| Headache | NR | 1 | NR | NR | NR | NR | **1** |
| Unstable Gait | NR | NR | NR | 1 | NR | NR | **1** |
| Gastric Discomfort | NR | NR | NR | 1 | NR | NR | **1** |
| Tremor | NR | NR | NR | 1 | NR | NR | **1** |
| Rash | NR | NR | 1 | 1 | NR | NR | **2** |
| Dyspepsia | NR | NR | NR | NR | NR | 1 | **1** |
| Mouth Dry | 9 | NR | NR | NR | NR | NR | **9** |
| Weakness | NR | NR | NR | NR | 3 | NR | **3** |
| Fever | NR | NR | NR | NR | 1 | NR | **1** |
| Constipation | NR | NR | 1 | NR | NR | NR | **1** |
| Other | 9 | NR | 5 | NR | 7 | NR | **21** |
| **Total AE** | **190** | **27** | **32** | **25** | **20** | **3** | **297** |

*NR; Not Reported

**Figure S5: Risk of bias**


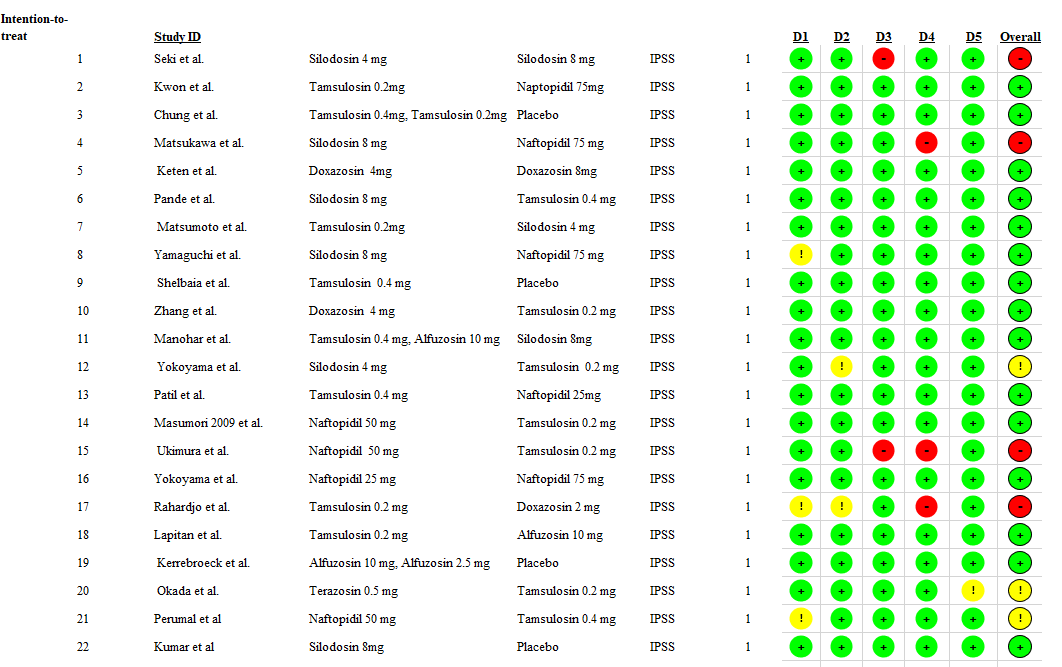
 **Risk of bias of included studies**


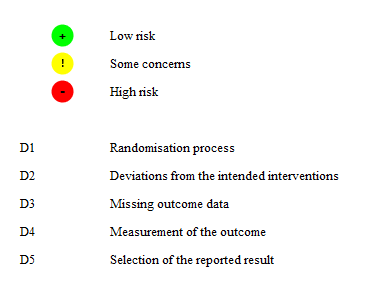


**GRADE assessment on the quality of evidence**

Within study bias, reporting bias, indirectness, imprecision, heterogeneity, and incoherence are the factors considered by the CINeMA framework.


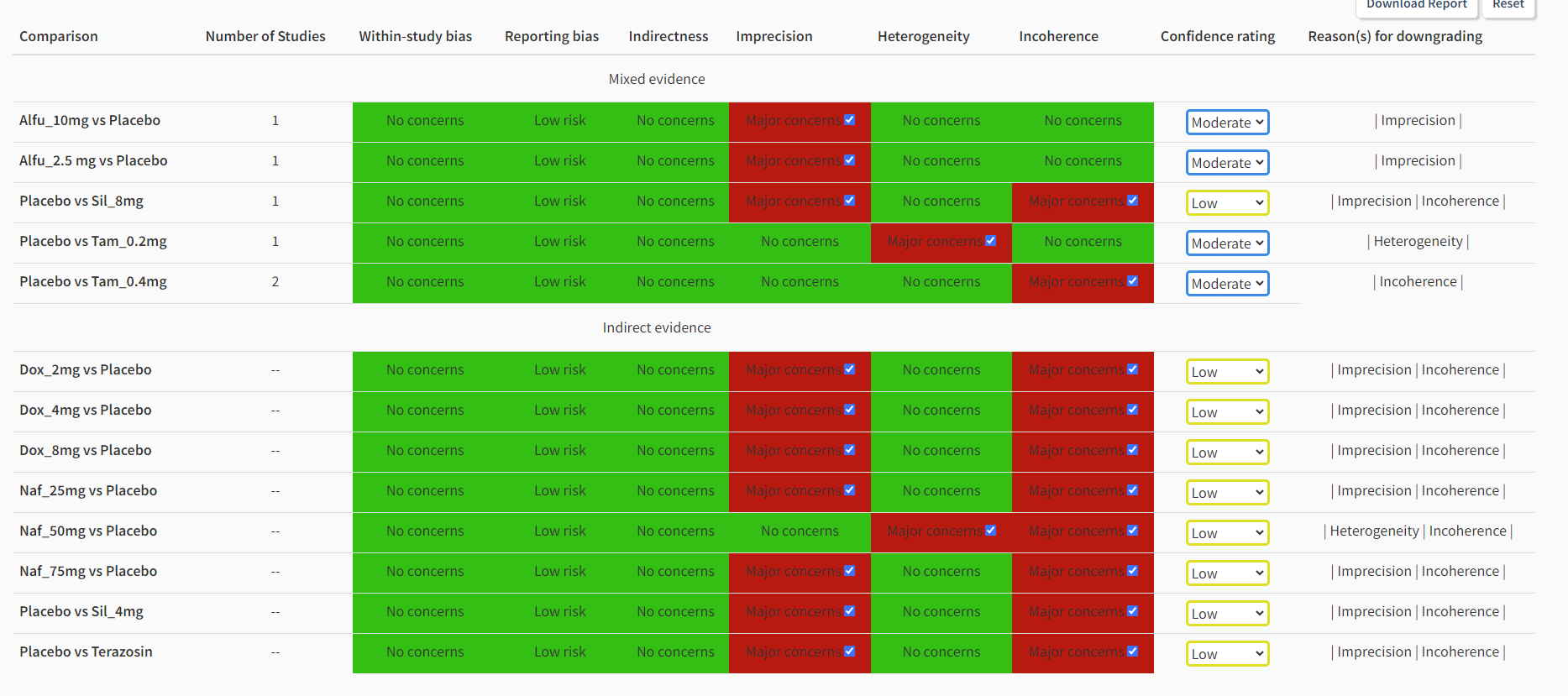
**Figure S6: GRADE rating for comparisons of IPSS.** Tam = tamsulosin, Alfu = alfuzosin, Naf = naftopidil, Tera = terazosin, Dox = doxazosin, Sil = silodosin.


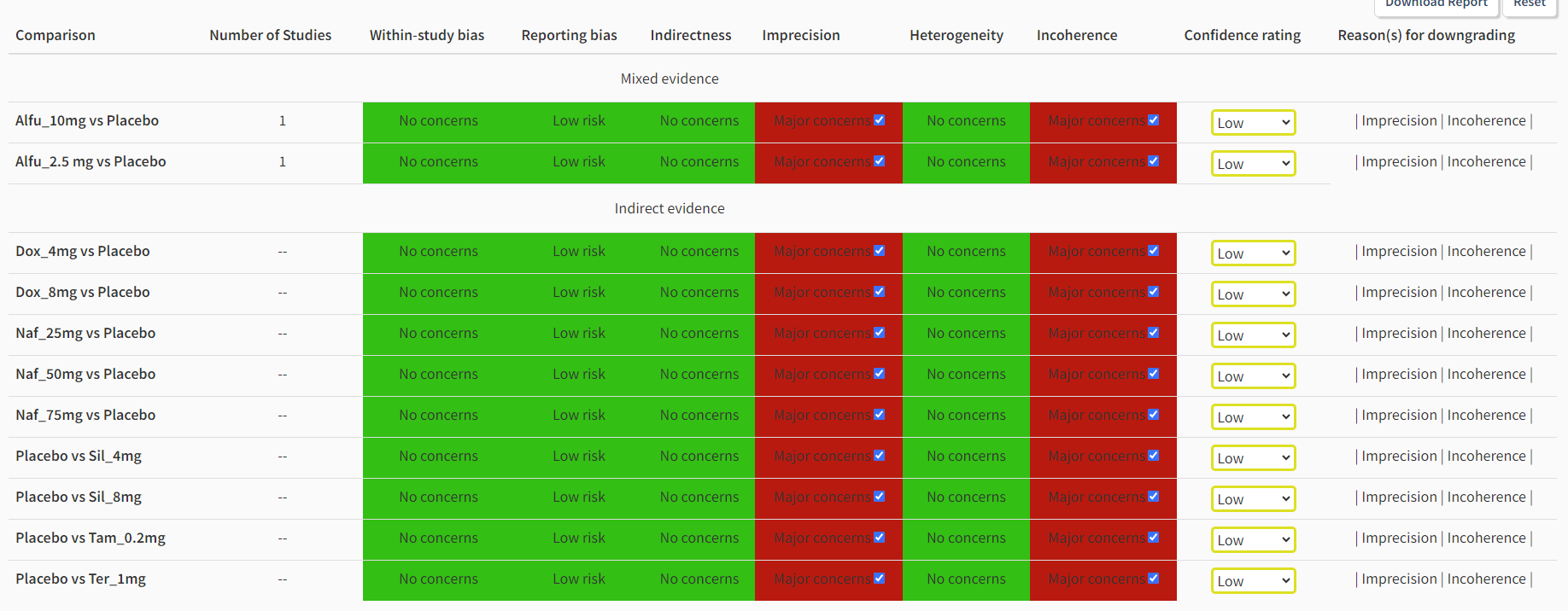
**Figure S7:** **GRADE rating for comparisons of QoL**.Tam = tamsulosin, Alfu = alfuzosin, Naf = naftopidil, Tera = terazosin, Dox = doxazosin, Sil = silodosin.


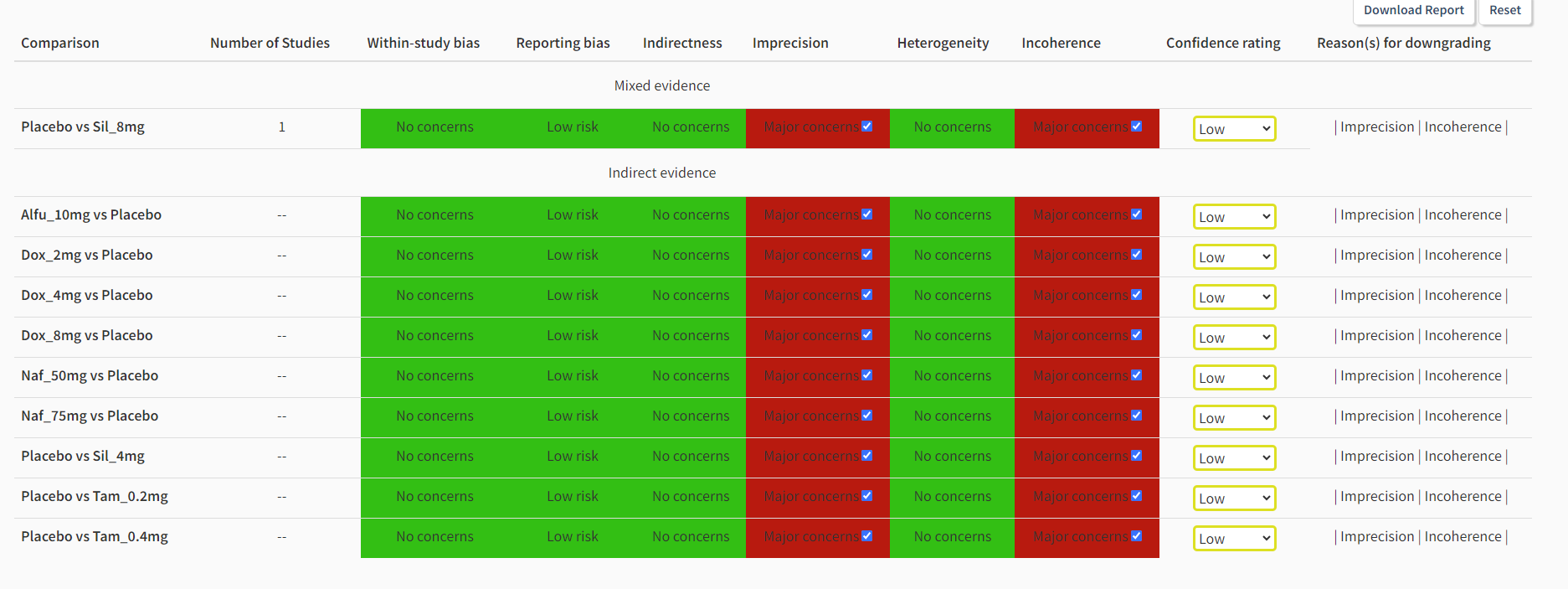


**Figure S8: GRADE rating for comparisons of PVR.** Tam = tamsulosin, Alfu = alfuzosin, Naf = naftopidil, Tera = terazosin, Dox = doxazosin, Sil = silodosin.


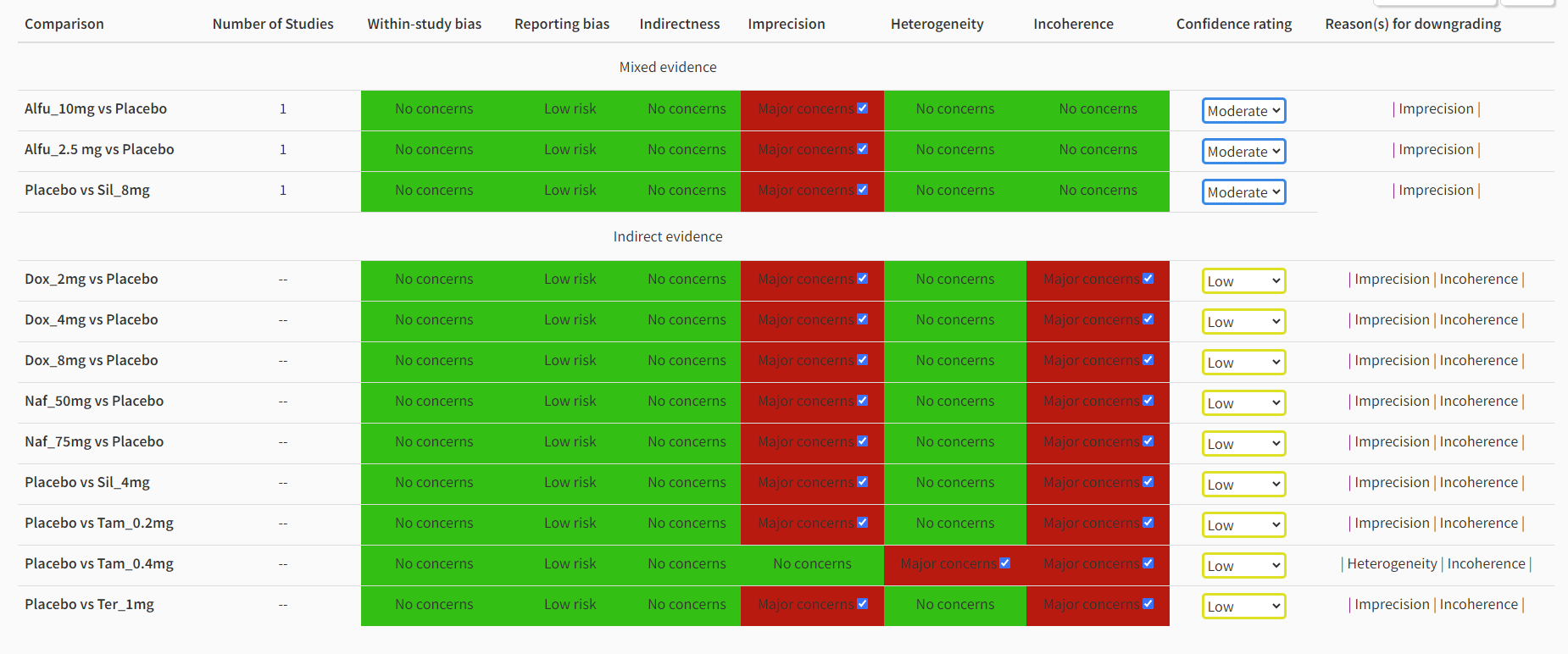
**Figure S9: GRADE rating for comparisons of Qmax.** Tam = tamsulosin, Alfu = alfuzosin, Naf = naftopidil, Tera = terazosin, Dox = doxazosin, Sil = silodosin.

**Table S9: Search strategy developed in various databases**

|  | **PubMed** |  |
| --- | --- | --- |
| **Search Number** | **Search Details** | **Results** |
| 15 | (("adrenergic alpha antagonists"[Pharmacological Action] OR "adrenergic alpha antagonists"[MeSH Terms] OR ("adrenergic"[All Fields] AND "alpha antagonists"[All Fields]) OR "adrenergic alpha antagonists"[All Fields] OR ("alpha"[All Fields] AND "blockers"[All Fields]) OR "alpha blockers"[All Fields] OR ("alfuzosin"[Supplementary Concept] OR "alfuzosin"[All Fields]) OR ("tamsulosin"[MeSH Terms] OR "tamsulosin"[All Fields] OR "tamsulosine"[All Fields]) OR ("doxazosin"[MeSH Terms] OR "doxazosin"[All Fields] OR "doxazosine"[All Fields]) OR ("terazosin"[Supplementary Concept] OR "terazosin"[All Fields] OR "terazosin"[All Fields] OR "terazosine"[All Fields]) OR ("silodosin"[Supplementary Concept] OR "silodosin"[All Fields] OR "silodosin s"[All Fields]) OR ("naftopidil"[Supplementary Concept] OR "naftopidil"[All Fields])) AND ("Benign prostatic hyperplasia"[All Fields] OR "BPH"[All Fields])) AND (clinicaltrial[Filter] OR controlledclinicaltrial[Filter] OR observationalstudy[Filter] OR randomizedcontrolledtrial[Filter]) | 553 |
| 14 | (("adrenergic alpha antagonists"[Pharmacological Action] OR "adrenergic alpha antagonists"[MeSH Terms] OR ("adrenergic"[All Fields] AND "alpha antagonists"[All Fields]) OR "adrenergic alpha antagonists"[All Fields] OR ("alpha"[All Fields] AND "blockers"[All Fields]) OR "alpha blockers"[All Fields] OR ("alfuzosin"[Supplementary Concept] OR "alfuzosin"[All Fields]) OR ("tamsulosin"[MeSH Terms] OR "tamsulosin"[All Fields] OR "tamsulosine"[All Fields]) OR ("doxazosin"[MeSH Terms] OR "doxazosin"[All Fields] OR "doxazosine"[All Fields]) OR ("terazosin"[Supplementary Concept] OR "terazosin"[All Fields] OR "terazosin"[All Fields] OR "terazosine"[All Fields]) OR ("silodosin"[Supplementary Concept] OR "silodosin"[All Fields] OR "silodosin s"[All Fields]) OR ("naftopidil"[Supplementary Concept] OR "naftopidil"[All Fields])) AND ("Benign prostatic hyperplasia"[All Fields] OR "BPH"[All Fields])) AND (clinicaltrial[Filter] OR observationalstudy[Filter] OR randomizedcontrolledtrial[Filter]) | 553 |
| 13 | (("adrenergic alpha antagonists"[Pharmacological Action] OR "adrenergic alpha antagonists"[MeSH Terms] OR ("adrenergic"[All Fields] AND "alpha antagonists"[All Fields]) OR "adrenergic alpha antagonists"[All Fields] OR ("alpha"[All Fields] AND "blockers"[All Fields]) OR "alpha blockers"[All Fields] OR ("alfuzosin"[Supplementary Concept] OR "alfuzosin"[All Fields]) OR ("tamsulosin"[MeSH Terms] OR "tamsulosin"[All Fields] OR "tamsulosine"[All Fields]) OR ("doxazosin"[MeSH Terms] OR "doxazosin"[All Fields] OR "doxazosine"[All Fields]) OR ("terazosin"[Supplementary Concept] OR "terazosin"[All Fields] OR "terazosin"[All Fields] OR "terazosine"[All Fields]) OR ("silodosin"[Supplementary Concept] OR "silodosin"[All Fields] OR "silodosin s"[All Fields]) OR ("naftopidil"[Supplementary Concept] OR "naftopidil"[All Fields])) AND ("Benign prostatic hyperplasia"[All Fields] OR "BPH"[All Fields])) AND (clinicaltrial[Filter]) | 553 |
| 12 | ("adrenergic alpha antagonists"[Pharmacological Action] OR "adrenergic alpha antagonists"[MeSH Terms] OR ("adrenergic"[All Fields] AND "alpha antagonists"[All Fields]) OR "adrenergic alpha antagonists"[All Fields] OR ("alpha"[All Fields] AND "blockers"[All Fields]) OR "alpha blockers"[All Fields] OR ("alfuzosin"[Supplementary Concept] OR "alfuzosin"[All Fields]) OR ("tamsulosin"[MeSH Terms] OR "tamsulosin"[All Fields] OR "tamsulosine"[All Fields]) OR ("doxazosin"[MeSH Terms] OR "doxazosin"[All Fields] OR "doxazosine"[All Fields]) OR ("terazosin"[Supplementary Concept] OR "terazosin"[All Fields] OR "terazosin"[All Fields] OR "terazosine"[All Fields]) OR ("silodosin"[Supplementary Concept] OR "silodosin"[All Fields] OR "silodosin s"[All Fields]) OR ("naftopidil"[Supplementary Concept] OR "naftopidil"[All Fields])) AND ("Benign prostatic hyperplasia"[All Fields] OR "BPH"[All Fields]) | 2,532 |
| 11 | "Benign prostatic hyperplasia"[All Fields] OR "BPH"[All Fields] | 20,438 |
| 10 | "adrenergic alpha antagonists"[Pharmacological Action] OR "adrenergic alpha antagonists"[MeSH Terms] OR ("adrenergic"[All Fields] AND "alpha antagonists"[All Fields]) OR "adrenergic alpha antagonists"[All Fields] OR ("alpha"[All Fields] AND "blockers"[All Fields]) OR "alpha blockers"[All Fields] OR ("alfuzosin"[Supplementary Concept] OR "alfuzosin"[All Fields]) OR ("tamsulosin"[MeSH Terms] OR "tamsulosin"[All Fields] OR "tamsulosine"[All Fields]) OR ("doxazosin"[MeSH Terms] OR "doxazosin"[All Fields] OR "doxazosine"[All Fields]) OR ("terazosin"[Supplementary Concept] OR "terazosin"[All Fields] OR "terazosin"[All Fields] OR "terazosine"[All Fields]) OR ("silodosin"[Supplementary Concept] OR "silodosin"[All Fields] OR "silodosin s"[All Fields]) OR ("naftopidil"[Supplementary Concept] OR "naftopidil"[All Fields]) | 13,795 |
| 9 | "BPH"[All Fields] | 66,205 |
| 8 | "Benign prostatic hyperplasia"[All Fields] | 14,959 |
| 7 | "naftopidil"[Supplementary Concept] OR "naftopidil"[All Fields] | 300 |
| 6 | "silodosin"[Supplementary Concept] OR "silodosin"[All Fields] OR "silodosin s"[All Fields] | 459 |
| 5 | "terazosin"[Supplementary Concept] OR "terazosin"[All Fields] OR "terazosin"[All Fields] OR "terazosin"[All Fields] | 954 |
| 4 | "doxazosin"[MeSH Terms] OR "doxazosin"[All Fields] OR "doxazosin"[All Fields] | 1,878 |
| 3 | "tamsulosin"[MeSH Terms] OR "tamsulosin"[All Fields] OR "tamsulosin"[All Fields] | 2,102 |
| 2 | "alfuzosin"[Supplementary Concept] OR "alfuzosin"[All Fields] | 611 |
| 1 | "adrenergic alpha antagonists"[Pharmacological Action] OR "adrenergic alpha antagonists"[MeSH Terms] OR ("adrenergic"[All Fields] AND "alpha antagonists"[All Fields]) OR "adrenergic alpha antagonists"[All Fields] OR ("alpha"[All Fields] AND "blockers"[All Fields]) OR "alpha blockers"[All Fields] | 65,216 |
|  | **Embase** |  |
| 11 | #1 AND #2 AND #10 | 887 |
| 10 | #3 OR #4 OR #5 OR #6 OR #7 OR #8 OR #9 | 333520 |
| 9 | ‘naftopidil’/exp OR naftopidil | 650 |
| 8 | ‘silodosin’/exp OR silodosin | 1188 |
| 7 | ‘doxazosin’/exp OR doxazosin | 8262 |
| 6 | ‘terazosin’/exp OR terazosin | 3941 |
| 5 | ‘alfuzosin’/exp OR alfuzosin | 2674 |
| 4 | ‘tamsulosin’/exp OR tamsulosin | 7236 |
| 3 | ‘alpha adrenergic receptor blocking agent’/exp OR ‘alpha adrenergic receptor blocking agent’ | 333260 |
| 2 | ‘prostate hypertrophy’/exp OR ‘prostate hypertrophy’ | 42458 |
| 1 | ‘randomized controlled trial’/exp OR ‘randomized controlled trial’ | 973160 |
|  | **Cochrane library** |  |
| 1 | MeSH descriptor: [Prostatic Hyperplasia] this term only | 2124 |
| 2 | MeSH descriptor: [Adrenergic Alpha-Antagonists] this term only | 1077 |
| 3 | MeSH descriptor: [Tamsulosin] this term only | 593 |
| 4 | MeSH descriptor: [Doxazosin] this term only | 398 |
| 5 | MeSH descriptor: [Adrenergic alpha-1 Receptor Antagonists] this term only | 317 |
| 6 | Naftopidil in Trials | 116 |
| 7 | Alfuzosin in Trials | 260 |
| 8 | Terazosin in Trials | 283 |
| 9 | Silodosin in Trials | 253 |
| 10 | #2 OR #5 in Trials | 1322 |
| 11 | #3 OR #4 OR #6 OR #6 OR #7 OR #8 OR #9 | 1706 |
| 12 | #1 AND #10 AND #11 with Cochrane Library publication date Between Jan 2000 and Jan 2023, in Trials | 244 |
|  | **Ovid MEDLINE** |  |
| 1 | exp Prostatic Hyperplasia/ | 23967 |
| 2 | exp Adrenergic alpha-Antagonists/ | 53722 |
| 3 | exp Adrenergic alpha-1 Receptor Antagonists/ | 15953 |
| 4 | exp Tamsulosin/ | 1426 |
| 5 | alfuzosin.mp. | 636 |
| 6 | silodosin.mp. | 484 |
| 7 | naftopidil.mp. | 307 |
| 8 | terazosin.mp. | 979 |
| 9 | exp Doxazosin/ | 1332 |
| 10 | 2 or 3 53722 | 53722 |
| 11 | 4 or 5 or 6 or 7 or 8 | 4388 |
| 12 | 1 and 10 and 11 | 1335 |

**Table S10: List of studies excluded at full-text screening stage, with brief reasons.**

| **Author** | **Year** | **Title** | **Reason for exclusion** |
| --- | --- | --- | --- |
|  | 2000 | Safety, efficacy and impact on Patients' quality of life of a long-term treatment with the alpha(1)-blocker alfuzosin in symptomatic patients with BPH. The Italian Alfuzosin Co-Operative Group | Irrelevant study design |
| Andersen et al | 2000 | Double-blind trial of the efficacy and tolerability of doxazosin in the gastrointestinal therapeutic system, doxazosin standard, and placebo in patients with benign prostatic hyperplasia | Outcome measure different |
| Curtis et al | 2000 | Single dose methodology to assess the influence of an alpha1-adrenoceptor antagonist on uroflowmetric parameters in patients with benign prostatic hyperplasia | Irrelevant study design |
| Feourcade et al | 2000 | [Efficiency and tolerance of terazosine in ambulatory patients with benign prostatic hypertrophy: comparative randomized and double-blind trial versus alfuzosin. The MG Terazosine Group] | Other language |
| Gratzke et al | 2000 | [Doxazosin in the gastrointestinal therapeutic system (GITS) and doxazosin standard in patients with benign prostatic hyperplasia. Double-blind trial of efficacy and tolerability] | Other language |
| Chapado et al | 2000 | Safety and efficacy of sustained-release alfuzosin on lower urinary tract symptoms suggestive of benign prostatic hyperplasia in 3,095 Spanish patients evaluated during general practice | Irrelevant study design |
| Chapado et al | 2000 | The clinical uroselectivity of alfuzosin is not significantly affected by the age of patients with lower urinary tract symptoms suggestive of benign prostatic hyperplasia | Irrelevant study design |
| Serment et al | 2000 | [Patterns of use of terazosine in current medical practice in ambulatory patients with obstructive and irritative obstructive disorders of urination] | Other language |
| Sujii et al | 2000 | Comparison of prazosin, terazosin and tamsulosin in the treatment of symptomatic benign prostatic hyperplasia: a short-term open, randomized multicenter study. BPH Medical Therapy Study Group. Benign prostatic hyperplasia | Outcome measure different |
| Roehrborn et al | 2001 | Efficacy and safety of once-daily alfuzosin in the treatment of lower urinary tract symptoms and clinical benign prostatic hyperplasia: a randomized, placebo-controlled trial | Outcome measure different |
| Suzuki et al | 2001 | [A randomized comparative study assessing once versus twice a day treatment of benign prostatic hyperplasia with terazosin] | Other language |
| Kerrebroeck et al | 2001 | The efficacy and safety of a new once-a-day formulation of an alpha-blocker | Irrelevant study design |
| Batista et al | 2002 | Tamsulosin: effect on quality of life in 2740 patients with lower urinary tract symptoms managed in real-life practice in Spain | Irrelevant study design |
| Hayashi et al | 2002 | [A comparative study assessing clinical effects of naftopidil and tamsulosin hydrochloride on benign prostatic hyperplasia] | Other language |
| Ju et al | 2002 | [The clinical efficacy of Naftopidil tablet in the treatment of benign prostatic hyperplasia] | Other language |
| Mimata et al | 2002 | Clinical characteristics of alpha-blocker responders in men with benign prostatic hyperplasia | Irrelevant outcome |
| Kerrebroeck et al | 2002 | Long-term safety and efficacy of a once-daily formulation of alfuzosin 10 mg in patients with symptomatic benign prostatic hyperplasia: open-label extension study | Outcome measure different |
| Ikemoto et al | 2003 | Usefulness of tamsulosin hydrochloride and naftopidil in patients with urinary disturbances caused by benign prostatic hyperplasia: a comparative, randomized, two-drug crossover study | Irrelevant study design |
| Kirby et al | 2003 | A randomized, double-blind crossover study of tamsulosin and controlled-release doxazosin in patients with benign prostatic hyperplasia | Irrelevant study design |
| Marks et al | 2003 | First dose efficacy of alfuzosin once daily in men with symptomatic benign prostatic hyperplasia | Irrelevant study design |
| Sugino et al | 2003 | [Clinical effects of naftopidil on nocturia associated with benign prostatic hyperplasia] | Other language |
| Ichioka et al | 2004 | Long-term treatment outcome of tamsulosin for benign prostatic hyperplasia | Irrelevant outcome |
| Kirby et al | 2004 | Doxazosin controlled release vs tamsulosin in the management of benign prostatic hyperplasia: an efficacy analysis | Outcome measure different |
| Samli et al | 2004 | Terazosin and doxazosin in the treatment of BPH: results of a randomized study with crossover in non-responders | Irrelevant study design |
| Chung et al | 2005 | Doxazosin for benign prostatic hyperplasia: an open-label, baseline-controlled study in Korean general practice | Outcome measure different |
| Gotoh et al | 2005 | Comparison of tamsulosin and naftopidil for efficacy and safety in the treatment of benign prostatic hyperplasia: a randomized controlled trial | Outcome measure different |
| Hernandez et al | 2005 | Controlled-release doxazosin in the treatment of benign prostatic hyperplasia | Irrelevant study design |
| Kirby et al | 2005 | Efficacy of extended-release doxazosin and doxazosin standard in patients with concomitant benign prostatic hyperplasia and sexual dysfunction | Irrelevant outcome |
| Muzzonigro et al | 2005 | Tamsulosin in the treatment of LUTS/BPH: an Italian multicentre trial | Outcome measure different |
| Nordling et al | 2005 | Efficacy and safety of two doses (10 and 15 mg) of alfuzosin or tamsulosin (0.4 mg) once daily for treating symptomatic benign prostatic hyperplasia | Outcome measure different |
| Roehrborn et al | 2005 | A double-blind placebo-controlled study evaluating the onset of action of doxazosin gastrointestinal therapeutic system in the treatment of benign prostatic hyperplasia | Outcome measure different |
| Trapeznikova et al | 2005 | [Clinical efficacy and safety of terazosine (setegis) in patients with benign prostatic hyperplasia with concomitant cardiovascular diseases] | Other language |
| Kawabe et al | 2006 | Silodosin, a new alpha1A-adrenoceptor-selective antagonist for treating benign prostatic hyperplasia: results of a phase III randomized, placebo-controlled, double-blind study in Japanese men | Outcome measure different |
| Nishino et al | 2006 | Comparison of two alpha1-adrenoceptor antagonists, naftopidil and tamsulosin hydrochloride, in the treatment of lower urinary tract symptoms with benign prostatic hyperplasia: a randomized crossover study | Irrelevant study design |
| Pompeo et al | 2006 | A randomised, double-blind study comparing the efficacy and tolerability of controlled-release doxazosin and tamsulosin in the treatment of benign prostatic hyperplasia in Brazil | Outcome measure different |
| Roehrborn et al | 2006 | Alfuzosin 10 mg once daily prevents overall clinical progression of benign prostatic hyperplasia but not acute urinary retention: results of a 2-year placebo-controlled study | Outcome measure different |
| Zhang et al | 2006 | [Sexual function of men with symptomatic benign prostatic hyperplasia and effect of Tamsulosin] | Other language |
| Yaycioglu et al | 2007 | The effects of two systemic alpha1-adrenergic blockers on pupil diameter: a prospective randomized single-blind study | Irrelevant outcome |
| Momose et al | 2007 | Crossover comparison study on the therapeutic effects of tamsulosin hydrochloride and naftopidil in lower urinary tract symptoms associated with benign prostatic hyperplasia | Irrelevant study design |
| Resnick et al | 2007 | Rapid onset of action with alfuzosin 10 mg once daily in men with benign prostatic hyperplasia: a randomized, placebo-controlled trial | Irrelevant outcome |
| Xue et al | 2007 | Doxazosin gastrointestinal therapeutic system versus tamsulosin for the treatment of benign prostatic hyperplasia: a study in Chinese patients | Irrelevant outcome |
| Yoshida et al | 2007 | Effect of tamsulosin hydrochloride on lower urinary tract symptoms and quality of life in patients with benign prostatic hyperplasia. Evaluation using bother score | Irrelevant outcome |
| Awa et al | 2008 | Clinical effect of alpha 1D/A adrenoceptor inhibitor naftopidil on benign prostatic hyperplasia: an international prostate symptom score and King's Health Questionnaire assessment | Irrelevant outcome |
| Ge et al | 2008 | [Effectiveness and safety of naftopidil for benign prostatic hyperplasia patients with overactive bladder symptoms] | Other language |
| Oh-oka et al | 2008 | Effect of naftopidil on nocturia after failure of tamsulosin | Irrelevant study design |
| Takao et al | 2008 | Early efficacy of silodosin in patients with lower urinary tract symptoms suggestive of benign prostatic hyperplasia | Irrelevant study design |
| Chung et al | 2009 | Sexuality and the management of BPH with alfuzosin (SAMBA) trial | Irrelevant outcome |
| Marks et al | 2009 | Silodosin in the treatment of the signs and symptoms of benign prostatic hyperplasia: a 9-month, open-label extension study | Irrelevant study design |
| Hanyu et al | 2010 | [A randomized controlled study comparing clinical effects of naftopidil and tamsulosin on benign prostatic hyperplasia] | Other language |
| Kim et al | 2010 | An open, non-comparative, multicentre study on the impact of alfuzosin on sexual function using the Male Sexual Health Questionnaire in patients with benign prostate hyperplasia | Irrelevant study design |
| Komiya et al | 2010 | Clinical effect of naftopidil on the quality of life of patients with lower urinary tract symptoms suggestive of benign prostatic hyperplasia: a prospective study | Irrelevant study design |
| Leungwattanakij et al | 2010 | Sexuality and management of benign prostatic hyperplasia with alfuzosin: SAMBA Thailand | Irrelevant outcome |
| Miyakita et al | 2010 | Short-term effects of crossover treatment with silodosin and tamsulosin hydrochloride for lower urinary tract symptoms associated with benign prostatic hyperplasia | Irrelevant study design |
| Sun et al | 2010 | Efficacy and safety of the doxazosin gastrointestinal therapeutic system for the treatment of benign prostate hyperplasia | Irrelevant study design |
| Yamanishi et al | 2010 | Urodynamic effects of silodosin, a new alpha 1A-adrenoceptor selective antagonist, for the treatment of benign prostatic hyperplasia | Irrelevant outcome |
| Chapple et al | 2011 | Silodosin therapy for lower urinary tract symptoms in men with suspected benign prostatic hyperplasia: results of an international, randomized, double-blind, placebo- and active-controlled clinical trial performed in Europe | Outcome measure different |
| Chung et al | 2011 | Comparative rapid onset of efficacy between doxazosin gastrointestinal therapeutic system and tamsulosin in patients with lower urinary tract symptoms from benign prostatic hyperplasia: a multicentre, prospective, randomised study | Outcome measure different |
| Funahashi et al | 2011 | Clinical efficacy of a loading dose of naftopidil for patients with benign prostate hyperplasia | Irrelevant study design |
| Kang et al | 2011 | Long-term outcome of tamsulosin for patients with lower urinary tract symptoms according to the treatment response defined by lower urinary tract symptom outcomes score | Irrelevant study design |
| Karadağ et al | 2011 | Randomized crossover comparison of tamsulosin and alfuzosin in patients with urinary disturbances caused by benign prostatic hyperplasia | Irrelevant study design |
| Roehrborn et al | 2011 | Symptomatic and urodynamic responses in patients with reduced or no seminal emission during silodosin treatment for LUTS and BPH | Irrelevant study design |
| Sakai et al | 2011 | [Efficacy of naftopidil in patients with overactive bladder associated with benign prostatic hyperplasia: prospective randomized controlled study to compare differences in efficacy between morning and evening medication] | Other language |
| Song et al | 2011 | The long-term effect of alfuzosin in patients with lower urinary tract symptoms suggestive of benign prostate hyperplasia: evaluation of voiding and storage function with respect to bladder outlet obstruction grade and contractility | Irrelevant study design |
| Sun et al | 2011 | Long-term efficacy and safety of tamsulosin hydrochloride for the treatment of lower urinary tract symptoms associated with benign prostatic hyperplasia: data from China | Irrelevant study design |
| Watanabe et al | 2011 | A randomized crossover study comparing patient preference for tamsulosin and silodosin in patients with lower urinary tract symptoms associated with benign prostatic hyperplasia | Irrelevant study design |
| Chapple et al | 2012 | [Silodosin therapy for lower urinary tract symptoms in men with suspected benign prostatic hyperplasia: results of an international, randomized, double-blind, placebo- and active-controlled clinical trial performed in Europe] | Outcome measure different |
| Kim et al | 2012 | Treatment satisfaction with low-dose tamsulosin for symptomatic benign prostatic hyperplasia: results from a multicentre cross-sectional survey | Irrelevant study design |
| Kojima et al | 2012 | Tamsulosin reduces nighttime urine production in benign prostatic hyperplasia patients with nocturnal polyuria: a prospective open-label long-term study using frequency-volume chart | Irrelevant study design |
| Masuda et al | 2012 | [Comparison of naftopidil and silodosin in the treatment of male lower urinary tract symptoms associated with benign prostatic hyperplasia: a randomized, crossover study] | Irrelevant study design |
| Akin et al | 2013 | The effect of first dose of tamsulosin on flow rate and its predictive ability on the improvement of LUTS in men with BPH in the mid-term | Irrelevant study design |
| Araki et al | 2013 | Comparison of 7 α(1)-adrenoceptor antagonists in patients with lower urinary tract symptoms associated with benign prostatic hyperplasia:a short-term crossover study | Irrelevant study design |
| Chung et al | 2013 | Evaluation of patient outcome after discontinuation of alfuzosin treatment for benign prostatic hyperplasia: a multicentre, prospective study | Irrelevant study design |
| Masumori et al | 2013 | α1-blocker tamsulosin as initial treatment for patients with benign prostatic hyperplasia: 5-year outcome analysis of a prospective multicenter study | Irrelevant study design |
| Shirakawa et al | 2013 | Silodosin versus naftopidil in Japanese patients with lower urinary tract symptoms associated with benign prostatic hyperplasia: a randomized multicenter study | Outcome measure different |
| Yamaguchi et al | 2013 | α1D/A-adrenoceptor antagonist naftopidil for the male lower urinary tract symptoms associated with benign prostatic hyperplasia: efficacy of dose increase therapy | Irrelevant study design |
| Descazeaud et al | 2014 | [Evolution of lower urinary tract symptoms under silodosin: factors influencing patients' satisfaction] | Irrelevant study design |
| Kim et al | 2014 | Efficacy and tolerability of tamsulosin 0.4 mg in Asian patients with lower urinary tract symptoms secondary to benign prostatic hyperplasia refractory to tamsulosin 0.2 mg: a randomized placebo controlled trial | Outcome measure different |
| Chung et al | 2015 | The preventive effect of tamsulosin on voiding dysfunction after prostate biopsy: a prospective, open-label, observational study | Irrelevant study design |
| Matsukawa et al | 2015 | A Slow Stream Is Pathophysiologically Related to a Poor Response to α1-Adrenoceptor Therapy in the Treatment of Storage Symptoms Associated With Benign Prostatic Hyperplasia | Irrelevant outcome |
| Salinas et al | 2015 | [Clinical and urodynamic results of alpha-blocker drug treatment in patients with lower urinary tract symptoms and benign prostatic hyperplasia] | Irrelevant study design |
| Montorsi et al | 2016 | Effectiveness and safety of silodosin in the treatment of lower urinary tract symptoms in patients with benign prostatic hyperplasia: A European phase IV clinical study (SiRE study) | Irrelevant study design |
| Takeshita et al | 2016 | Randomized Crossover Comparison of the Short-Term Efficacy and Safety of Single Half-Dose Silodosin and Tamsulosin Hydrochoride in Men With Lower Urinary Tract Symptoms Secondary to Benign Prostatic Hyperplasia | Irrelevant study design |
| Chung et al | 2017 | Clinical Efficacy and Safety of Naftopidil Treatment for Patients with Benign Prostatic Hyperplasia and Hypertension: A Prospective, Open-Label Study | Irrelevant study design |
| Cho et al | 2018 | Evaluating the Efficacy and Safety of Silodosin on Nocturia in Patients With Benign Prostatic Hyperplasia: A Multicenter, Prospective, Open-label, Single-arm, Phase IV Trial | Irrelevant study design |
| Ichihara et al | 2018 | Silodosin as second-line α-blocker monotherapy in patients with benign prostatic hyperplasia: A prospective observational study | Irrelevant study design |
| Topazio et al | 2018 | Intravescical prostatic protrusion is a predictor of alpha blockers response: results from an observational study | Irrelevant study design |
| Xue et al | 2018 | The evaluation of nocturia in patients with lower urinary tract symptoms suggestive of benign prostatic hyperplasia and the analysis of the curative effect after medical or placebo therapy for nocturia: a randomized placebo-controlled study | Irrelevant outcome |
| Yang et al | 2018 | An open-label, prospective interventional study of the tolerability and efficacy of 0.4 mg oral tamsulosin oral controlled absorption system in men with lower urinary tract symptoms associated with benign prostatic hyperplasia who are unsatisfied with treatment with 0.2 mg tamsulosin | Irrelevant comparator |
| Takahashi et al | 2019 | Post-Marketing Surveillance of Silodosin in Patients with Benign Prostatic Hyperplasia and Poor Response to Existing Alpha-1 Blockers: The SPLASH Study | Irrelevant study design |
| Abrams et al | 1997 | A dose-ranging study of the efficacy and safety of tamsulosin, the first prostate-selective α(1A)-adrenoceptor antagonist, in patients with benign prostatic obstruction (symptomatic benign prostatic hyperplasia) | Irrelevant outcome |
| Ariwicaksono | 2019 | Efficacy of silodosin vs tamsulosin in patients with benign prostatic hyperplasia: A double-blind randomized clinical trial | Outcome measure different |
| Buzelin et al | 1997 | Comparison of tamsulosin with alfuzosin in the treatment of patients with lower urinary tract symptoms suggestive of bladder outlet obstruction (symptomatic benign prostatic hyperplasia) | Outcome measure different |
| Chapple et al | 2011 | Silodosin therapy for lower urinary tract symptoms in men with suspected benign prostatic hyperplasia: Results of an international, randomized, double-blind, placebo- and active-controlled clinical trial performed in Europe | Outcome measure different |
| Choo et al | 2014 | Safety and efficacy of 8-mg once-daily vs 4-mg twice-daily silodosin in patients with lower urinary tract symptoms suggestive of benign prostatic hyperplasia (silver study): A 12-week, double-blind, randomized, parallel, multicenter study | Irrelevant comparator |
| Debruyne et al | 1996 | The international terazosin trial: A multicentre study of the long-term efficacy and safety of terazosin in the treatment of benign prostatic hyperplasia | population |
| Gittelman et al | 2011 | Effect of silodosin on specific urinary symptoms associated with benign prostatic hyperplasia: Analysis of international prostate symptom scores in 2 phase III clinical studies | Outcome measure different |
| Homma et al | 2010 | Ejaculation disorder is associated with increased efficacy of silodosin for benign prostatic hyperplasia | Outcome measure different |
| Kawabe et al | 2006 | Silodosin, a new α1A-adrenoceptor-selective antagonist for treating benign prostatic hyperplasia: Results of a phase III randomized, placebo-controlled, double-blind study in Japanese men | Outcome measure different |
| Kim et al | 2014 | Efficacy and tolerability of tamsulosin 0.4mg in Asian patients with lower urinary tract symptoms secondary to benign prostatic hyperplasia refractory to tamsulosin 0.2mg: A randomized placebo controlled trial | statistically incorrect |
| Lee et al | 1997 | Clinical comparison of selective and non-selective α1A-adrenoreceptor antagonists in benign prostatic hyperplasia: Studies on tamsulosin in a fixed dose and terazosin in increasing doses | Outcome measure different |
| Lee et al | 2012 | Efficacy and safety of tamsulosin for treating lower urinary tract symptoms associated with benign prostatic hyperplasia: A multicenter, randomized, controlled, open-label non-inferiority study | Outcome measure different |
| Lepor et al | 1998 | Phase III multicenter placebo-controlled study of tamsulosin in benign prostatic hyperplasia | Outcome measure different |
| Gittelman et al | 2010 | Effect of silodosin on specific urinary symptoms associated with benign prostatic hyperplasia: analysis of international prostate symptom scores in 2 phase iii clinical studies | Irrelevant study design |
| Marks et al | 2008 | Rapid efficacy of the highly selective α1A-adrenoceptor antagonist silodosin in men with signs and symptoms of benign prostatic hyperplasia: Pooled results of 2 phase 3 studies | Outcome measure different |
| Matsukawa et al | 2019 | What Are Factors Contributing to Improvement of Overactive Bladder Symptoms after Alpha-1 Blocker Treatment in Patients with Both Storage and Voiding Symptoms? | Outcome measure different |
| Narayan et al | 2000 | A comparison of two phase III multicenter, placebo-controlled studies of tamsulosin in BPH | Outcome measure different |
| Özbey et al | 1999 | Effects of doxazosin in men with benign prostatic hyperplasia: Urodynamic assessment | Outcome measure different |
| Pompeo et al | 2006 | A randomised, double-blind study comparing the efficacy and tolerability of controlled-release doxazosin and tamsulosin in the treatment of benign prostatic hyperplasia in Brazil | Outcome measure different |
| Resnick et al | 2007 | Rapid onset of action with alfuzosin 10 mg once daily in men with benign prostatic hyperplasia: A randomized, placebo-controlled trial | Outcome measure different |
| Roehrborn et al | 2001 | Efficacy and safety of once-daily alfuzosin in the treatment of lower urinary tract symptoms and clinical benign prostatic hyperplasia: A randomized, placebo-controlled trial | Outcome measure different |
| Roehrborn et al | 2006 | Alfuzosin 10 mg once daily prevents overall clinical progression of benign prostatic hyperplasia but not acute urinary retention: Results of a 2-year placebo-controlled study | Outcome measure different |
| Roehrborn et al | 2005 | A double-blind placebo-controlled study evaluating the onset of action of doxazosin gastrointestinal therapeutic system in the treatment of benign prostatic hyperplasia | Outcome measure different |
| Rosen et al | 2007 | Effects of alfuzosin 10 mg once daily on sexual function in men treated for symptomatic benign prostatic hyperplasia | Outcome not complete |
| Shirakawa et al | 2013 | Silodosin versus naftopidil in japanese patients with lower urinary tract symptoms associated with benign prostatic hyperplasia: A randomized multicenter study | methodology not clear |
| Singh et al | 2012 | Efficacy and safety of tamsulosin (0.4 mg) once daily for treating symptomatic benign prostatic hyperplasia | full text not available |
| Tanaka et al | 2015 | Efficacy of naftopidil for nocturia in male patients with lower urinary tract symptoms: Comparison of morning and evening dosing | Outcome measure different |
| Tsujii et al | 2000 | Comparison of prazosin, terazosin and tamsulosin in the treatment of symptomatic benign prostatic hyperplasia: A short-term open, randomized multicenter study | Outcome measure different |
| Kerrebroeck et al | 2002 | Long-term safety and efficacy of a once-daily formulation of alfuzosin 10 mg in patients with symptomatic benign prostatic hyperplasia: Open-label extension study | duration different, extention study |
| Xue et al | 2007 | Doxazosin gastrointestinal therapeutic system versus tamsulosin for the treatment of benign prostatic hyperplasia: A study in Chinese patients | Outcome not complete |
| Yu et al | 2011 | Non-inferiority of silodosin to tamsulosin in treating patients with lower urinary tract symptoms (LUTS) associated with benign prostatic hyperplasia (BPH) | Outcome measure different |

**Appendix S1: Details on Inconsistency**

**IPSS Global approach**
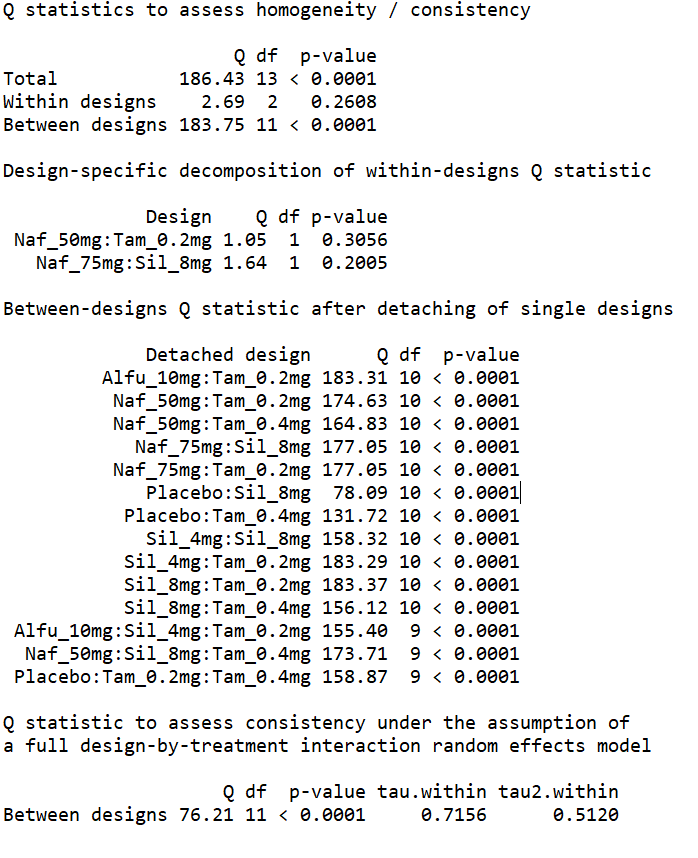


**QoL Global Approach**


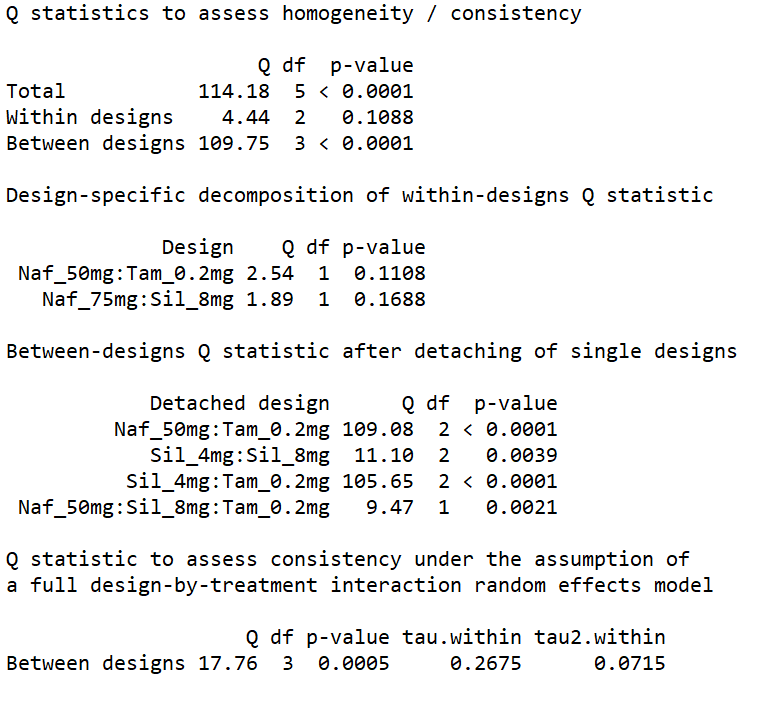


**PVR Global Approach**


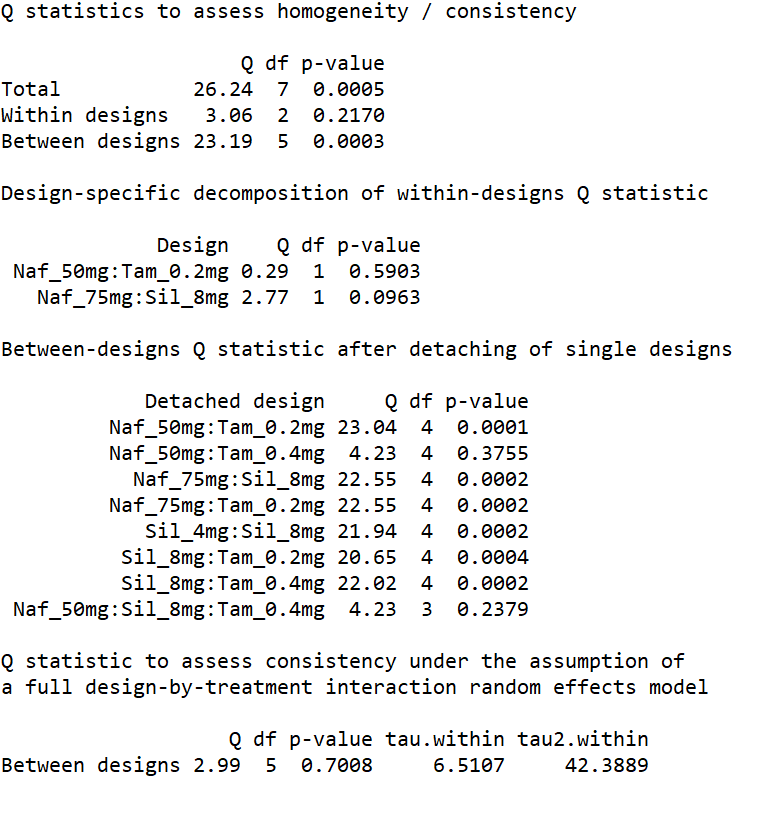


**Qmax Global Approach**


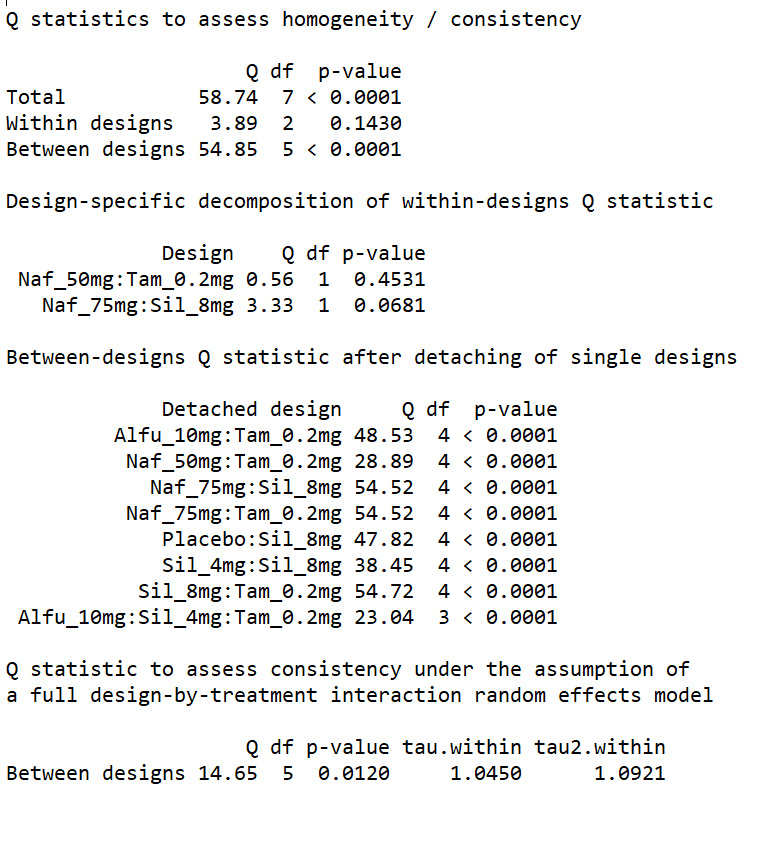

Supplement: Supplementary file 1 — Supplementary Information. [file 41598_2024_61977_MOESM1_ESM.docx]
